# Comparing De Novo Genome Assembly: The Long and Short of It

**DOI:** 10.1371/journal.pone.0019175

**Published:** 2011-04-29

**Authors:** Giuseppe Narzisi, Bud Mishra

**Affiliations:** 1 Courant Institute of Mathematical Sciences, New York University, New York, New York, United States of America; 2 NYU School of Medicine, New York University, New York, New York, United States of America; University of Leuven, Belgium

## Abstract

Recent advances in DNA sequencing technology and their focal role in Genome Wide Association Studies (GWAS) have rekindled a growing interest in *the whole-genome sequence assembly (WGSA) problem*, thereby, inundating the field with a plethora of new formalizations, algorithms, heuristics and implementations. And yet, scant attention has been paid to comparative assessments of these assemblers' quality and accuracy. No commonly accepted and standardized method for comparison exists yet. Even worse, widely used metrics to compare the assembled sequences emphasize only size, poorly capturing the contig quality and accuracy. This paper addresses these concerns: it highlights common anomalies in assembly accuracy through a rigorous study of several assemblers, compared under both standard metrics (N50, coverage, contig sizes, etc.) as well as a more comprehensive metric (Feature-Response Curves, FRC) that is introduced here; FRC transparently captures the trade-offs between contigs' quality against their sizes. For this purpose, most of the publicly available major sequence assemblers – both for low-coverage long (Sanger) and high-coverage short (Illumina) reads technologies – are compared. These assemblers are applied to microbial (Escherichia coli, Brucella, Wolbachia, Staphylococcus, Helicobacter) and partial human genome sequences (Chr. Y), using sequence reads of various read-lengths, coverages, accuracies, and with and without mate-pairs. It is hoped that, based on these evaluations, computational biologists will identify innovative sequence assembly paradigms, bioinformaticists will determine promising approaches for developing “next-generation” assemblers, and biotechnologists will formulate more meaningful design desiderata for sequencing technology platforms. A new software tool for computing the FRC metric has been developed and is available through the AMOS open-source consortium.

## Introduction

Since the completion of the Herculean task of the Human Genome project (HGP) in 2003, the genomics community has witnessed a deluge of sequencing projects: They range from metagenomes, microbiomes, and genomes to transcriptomes; often, they focus on a multitude of organisms, populations and ecologies. In addition, the subsequent advent of high-throughput Gen-1, Gen-2 and Gen-3 of sequencing technologies – with their promise to considerably reduce the genome sequencing cost – now appear poised to usher in a personal genomics revolution [1]–[3]. In this context, the accuracy of the resulting reference genome sequences and their suitability for biomedical applications play a decisive role, as they additionally depend upon many parameters of the sequencing platforms: read lengths, base-calling errors, homo-polymer errors, etc. These parameters continue to change at a faster-and-faster pace, as the platform chemistry and engineering continue to evolve.

However, in the ensuing euphoria, what seems to have been left neglected is a constructive and critical retrospection, namely: (1) to appraise the strengths and weaknesses of the schemes, protocols and algorithms that now comprise a typical “sequencing pipeline”; (2) to scrutinize the accuracy and usefulness of the assembled sequences by any standard pipeline; and (3) to build technologically agnostic assembly algorithms that would easily adapt to the current fast evolving biotechnologies. Historically, all these sequence assemblers – each representing many man-years of effort – appear to require complete and costly overhaul, with each introduction of a new short-read or long-range technology.

This paper addresses these issues by presenting a diverse set of empirical studies, where, to ensure statistical significance of the results, a large number of sequence assemblers are evaluated in terms of assembly accuracy and performance: assemblers include those developed both for Sanger and next-generation (Gen-1) technologies, and evaluation is performed using both traditional metrics and new metrics. Specifically the paper is organized as follows: first, the study is motivated by a discussion of current state-of-the-art sequence assemblers, their underlying assembly paradigms and relations to various metrics; next standard assembly comparison and validation techniques are critically examined – especially in light of various anomalies they produce – highlighting the need for better quality assessment; then the experimental protocol is explained together with the benchmarks used in this study; assembly results and quality analysis are then presented using standard paired and unpaired, low- and high-coverage, long and short reads from previously collected real and simulated data; and finally a discussion of the presented results concludes the paper.

### Motivation: State-of-the-art Sequence Assemblers

In the most general setting, the *DNA sequence assembly problem*, as commonly referred, addresses the reconstruction of a DNA sequence from a collection of randomly sampled fragments. This process is further complicated by the presence of haplotypic ambiguities, sequencing errors and repetitive sections. Most assemblers are based on an intuitively obvious assumption: if two sequence reads (two strings on a four-letter alphabet, produced by the sequencing machine) share a common overlapping substring of letters, then it is because they *are likely to* have originated from the same chromosomal regions in the genome. The basic assumption, can be made more precise, by additionally taking into account the facts that the sequence reads could come from either Watson or Crick strand, and that if two strings are part of a mate-pair then the estimated distance between the reads imposes an additional constraint, not to be violated. Once such overlaps structures among the sequence reads are determined, the assembler places the reads in a lay-out and combines the reads together to create a consensus sequence–not unlike how one solves a complex jigsaw puzzle. Based on the underlying search strategies, most of the state-of-the-art assemblers belong practically to two major categories: *greedy* and *graph-based*.

#### Greedy

Computational biologists first formalized the shotgun sequence assembly problem in terms of an approximation to finding the shortest common superstring (SCS) of a set of sequences [4]. Because of its assumed computational intractability (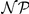-completeness), a large number of the available approaches for genome sequence assembly resorted to greedy methods. Greedy algorithms typically construct the solution incrementally using the following basic steps: (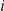) pick the highest scoring overlap; (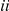) merge the two overlapping fragments and add the resulting new sequence to the pool of sequences; (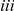) repeat until no more merges can be carried out. In addition, after each merge operation, the region of the overlay is heuristically corrected in some reasonable manner (whenever possible). Regions that fail to yield to these error-correction heuristics are relinquished as irrecoverable and shown as gaps. Mate-pairs information is used judiciously during the merging process to further validate the connection between the two sequences. At the end of this process a single solution (consisting of the set of assembled contigs) is generated as output. Well known assemblers in this category include: TIGR [5], PHRAP [6], CAP3 [7], PCAP [8] and Phusion [9].

#### Graph-based

Graph-based algorithms start by preprocessing the sequence-reads to determine the pair-wise overlap information and represent these binary relationships as (unweighted) edges in a string-graph. The problem of finding a consistent lay-out can then be formulated in terms of searching a collection of paths in the graph satisfying certain specific properties. Paths, computed thus, correspond to *contigs* (contiguous sequences of the genome, consistently interpreting disjoint subsets of sequence reads). Contingent upon how the overlap relation is represented in these graphs, two dominant assembly paradigms have emerged: *overlap-layout-consensus* (OLC) and *sequencing-by-hybridization* (SBH). In the OLC approach, the underlying graph (overlap graph) comprises nodes representing reads and edges representing overlaps. Ideally, the goal of the algorithm is to determine a simple path traversing all the nodes – that is, a Hamiltonian path. For a general graph, this problem is known to lead to an 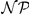-hard optimization problem [10] though polynomial-time solutions exist for certain specialized graphs (e.g., interval graphs, etc.) [11]. A popular heuristic strategy is typically employed as follows: (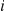) remove “contained” and “transitivity” edges; (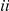) collapse “unique connector” overlaps (chordal subgraph with no conflicting edges) to compute the contigs; (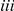) use mate-pairs to connect and order the contigs. Hence, the set of contigs, as computed thus, corresponds to the set of nonintersecting simple paths in the reduced graph. Well known assemblers in this category include: CELERA [12], CABOG [13], ARACHNE [14], Minimus [15] and Edena [16]. In the SBH (Sequencing by Hybridization) approach, the underlying graph encodes overlaps by nodes and the reads containing a specific overlap by edges incident to the corresponding node (for that overlap). This dual representation may be described in terms of the following steps: (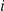) partition the reads into a collection of overlapping 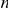-mers (an 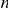-mer is a substring of length 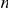); (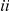) build a de Bruijn graph in which each edge is an 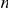-mer from this collection and the source and destination nodes are respectively the 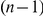-prefix and 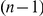-suffix of the corresponding 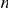-mer. In this new graph, instead of a Hamiltonian path, one may seek to find an Eulerian path, containing every edge exactly once; thus computed genome sequence would then provide a consistent explanation for every consecutive 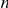-mers on any sequence read. More importantly, such a graph is linear in the size of the input and allows the computation of an Eulerian path to be carried out in linear time. Because of the succinct representation it generates, this approach has motivated many new algorithms for assembling short reads, which typically come with very high coverage. However, in practice, these algorithms do not directly compute Eulerian paths since many complications arise. First, sequencing errors in the read data introduce many spurious (false-positive) edges which mislead the algorithm. Second, for any reasonable choice of 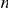, the size of the graph is dramatically bigger than the one in the overlap-layout-consensus strategy. Third, a de Bruijn graph may not have a unique Eulerian path, and an assembler must find a particular Eulerian path subject to certain extraneous constraints; thus, it must solve a somewhat general problem, namely *the Eulerian-superpath problem*: given an Eulerian graph and a sequence of paths, find an Eulerian path in the Eulerian graph that contains all these paths as sub-paths. It is known that finding the shortest Eulerian superpath is, unfortunately, also an 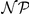-hard problem [10]. In practice, a heuristic method is used to compute such a superpath by applying a series of transformations to the original Eulerian graph. Prominent examples of the SBH approach include: Euler [17], Velvet [18], ABySS [19] and SOAPdenovo [20].

#### Seed-and-Extend

A new variation of greedy assemblers (specifically designed for short reads and based on a contig extension heuristic scheme) works with an efficient prefix-tree data-structure. In this framework a contig is elongated at either of its ends so long as there exist reads with a prefix of minimal length, provided that it perfectly matches an end of the contig. Example of assemblers belonging to this new category are: SHARCGS [21], SSAKE [22], QSRA [23] and Taipan [24].

#### Branch-and-Bound

In contrast to traditional graph based assemblers, a new sequence assembly method (i.e., B&B-based SUTTA [25]) has been more recently developed. It employs combinatorial optimization techniques typically used for other well-known 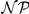-hard problems (satisfiability problem, traveling salesman problem, etc.). At a high level, SUTTA's framework views the assembly problem simply as that of constrained optimization: it relies on a rather simple and easily verifiable definition of feasible solutions as “consistent layouts. ” It generates potentially all possible consistent layouts, organizing them as paths in a “double-tree” structure, rooted at a randomly selected “seed” read. A path is progressively evaluated in terms of an optimality criteria, encoded by a set of score functions based on the set of overlaps along the lay-out. This strategy enables the algorithm to concurrently assemble and check the validity of the lay-outs (with respect to various long-range information) through well-chosen constraint-related penalty functions. Complexity and scalability problems are addressed by pruning most of the implausible lay-outs, using a *branch-and-bound* scheme. Ambiguities, resulting from repeats or haplotypic dissimilarities, may occasionally delay immediate pruning, forcing the algorithm to *lookahead*, but in practice, do not exact a high price in computational complexity of the algorithm.

Finally, all the techniques described earlier need to separately incorporate mate-pairs information as they play an important role in resolving repeats as well as in generating longer contigs, thus dramatically reducing the cost of the assembly-finishing. Note that although mate-pairs are typically expensive and slow to obtain, prone to statistical errors and frequently incapable of spanning longer repeat regions, historically, they have played an important role in the assembly of large genomes as other cheaper and more informative mapping techniques have not been widely available. In addition, they have become essential to many emerging sequencing technologies (454, Illumina-Solexa, etc.), which can generate very high coverage short reads data (from 30 bp up to 500 bp).

Table 1 presents a nearly exhaustive list of the major state-of-the-art sequence assemblers organized by assembly paradigms. It also contains the sequencing platform(s) that each assembler is capable of handling.

**Table 1:** List of sequence assemblers.

Name	Read Type	Algorithm	Reference
SUTTA	long & short	B&B	(Narzisi and Mishra [25], 2010)
ARACHNE	long	OLC	(Batzoglou et al. [14], 2002)
CABOG	long & short	OLC	(Miller et al. [13], 2008)
Celera	long	OLC	(Myers et al. [12], 2000)
Edena	short	OLC	(Hernandez et al. [16], 2008)
Minimus (AMOS)	long	OLC	(Sommer et al. [15], 2007)
Newbler	long	OLC	454/Roche
CAP3	long	Greedy	(Huang and Madan [7], 1999)
PCAP	long	Greedy	(Huang et al. [8], 2003)
Phrap	long	Greedy	(Green [6], 1996)
Phusion	long	Greedy	(Mullikin and Ning [9], 2003)
TIGR	long	Greedy	(Sutton et al. [5], 1995)
ABySS	short	SBH	(Simpson et al. [19], 2009)
ALLPATHS	short	SBH	(Butler et al. [46], [47], 2008/2011)
Euler	long	SBH	(Pevzner et al. [17], 2001)
Euler-SR	short	SBH	(Chaisson and Pevzner [35], 2008)
Ray	long & short	SBH	(Boisvert et al. [48], 2010)
SOAPdenovo	short	SBH	(Li et al. [20], 2010)
Velvet	long & short	SBH	(Zerbino and Birney [18], [49], 2008/2009)
PE-Assembler	short	Seed-and-Extend	(Ariyaratne and Sung [50], 2011)
QSRA	short	Seed-and-Extend	(Bryant et al. [23], 2009)
SHARCGS	short	Seed-and-Extend	(Dohm et al. [21], 2007)
SHORTY	short	Seed-and-Extend	(Hossain et al. [51], 2009)
SSAKE	short	Seed-and-Extend	(Warren et al. [22], 2007)
Taipan	short	Seed-and-Extend	(Schmidt et al. [24], 2009)
VCAKE	short	Seed-and-Extend	(Jeck et al. [52], 2007)

Reads are defined as “long” if produced by Sanger technology and “short” if produced by Illumina technology . Note that Velvet was designed for micro-reads (e.g. Illumina) but long reads can be given in input as additional data to resolve repeats in a greedy fashion.

### The need for Quality Assessment

Though validation and performance evaluation of an assembler are very important tasks, no commonly accepted and standardized method for this purpose exists currently. The genome validation process appears to have remained a largely manual and expensive process, with most of the genomes simply accepted as draft assemblies. For instance, the initial “draft” sequence of the human genome [26] has been revised several times, since its first publication, each revision eliminating various classes of errors through successive algorithmic advances; nevertheless, genome sequencing continues to be viewed as an inexact craft and inadequate in controlling the number of errors, which in the draft genomes are estimated to be up to hundred or even thousands [27]. The errors in such draft assemblies fall into several categories: collapsed repeats, rearrangements, inversions, etc., with their incidents, varying from genome to genome.

It should be noted that the most popular metrics for evaluating an assembly (e.g., contig size and N50), emphasize only size, poorly capturing the contig quality as they do not contain all the information needed to judge the correctness of the assembly. For example N50 is defined as the largest number 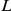 such that the combined length of all contigs of length 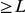 is at least 50% of the total length of all contigs. In these scenarios, an assembler that sacrifices assembly quality in exchange for contig sizes, appears to outperform others, despite generating consensus sequences replete with rearrangement errors. For example, in the extreme case, an assembly consisting of one large contig of roughly the size of the genome is useless if mis-assembled. On the other extreme, an assembly consisting of many short contigs covering only the inter-repeat regions of the genome could have very high accuracy although contigs might be too short to be used, for example, for gene-annotation efforts. Similarly, just a simple count of the number of mis-assembled contigs obtained by alignments to the reference genome (if available), a metric typically used to compare short-read assemblies, is not satisfying either, because it does not take into account the various structural properties of the contigs and of the reads contained in it. For example one single mis-assembled contig could represent the longest contig in the set and it could include multiple types of errors (mate-pair orientation, depth of coverage, polymorphism, etc) which should be weighted differently. Although the evaluation of the tradeoff between contig length and errors is an important problem, there is very little in the literature discussing this topic or applying it to evaluate the assembly quality of different assemblers. Responding to this need we have developed a new metric, Feature-Response curve (FRC), which captures the trade-offs between quality and contig size more accurately (see section Materials and Methods). The FRC shares many similarities with classical ROC (receiver-operating characteristic) curves, commonly employed to compare the performance of statistical inference procedures. Analogous to ROC, FRC emphasizes how well an assembler exploits the relation between incorrectly-assembled contigs (false positives, contributing to “features”) against gaps in assembly (false negatives, contributing to fraction of genome-coverage or “response”), when all other parameters (read-length, sequencing error, depth, etc.) are held constant. An example of FRC appears briefly in a recent paper that focuses on SUTTA's implementation and application to short reads [25]. However the current paper focuses on the FRC-based comparisons of a wider class of technologies and algorithms. Finally, although SUTTA was developed by us, as the authors of this comparison paper, we have strived as best as possible to adopt a style of full impartiality to avoid any bias in the discussions of the comparison results.

Through an empirical comparative analysis of the outputs of several assemblers, this paper is able to highlight various anomalies in assemblies produced under different strategies and approaches. The analyses are performed under both standard metrics as well as new metrics (FRC). In addition, visual inspection of the consistency of the assembled contigs is enabled by several graphic representations of the alignment against the reference genome, e.g., through dot plots.

### Experimental protocol and Benchmarking

Before discussing the results, we present the benchmark and assemblers that we have selected for comparison and explain the design of the experimental protocol adopted here.

### Benchmark selection

In evaluating the assembly quality of different assemblers, several criteria were used in choosing bench-mark data sets, assembly-pipelines and comparison metrics: e.g., statistical significance, ease of reproducibility, accessibility in public domain etc. For example, by avoiding expensive studies with large sized genome-assembly and specialized (but not widely available) technologies, we wished to ensure that the reported results could be widely reproduced, revalidated and extended – even by moderate-sized biology laboratories or small teams of computer scientists. To the extent possible, we have favored the use of real data over synthetic data.

Consequently, we have not included large genomes (e.g., whole haplotypic human genomes) or single-molecule technologies (PacBiosciences or Optical Mapping), but ensured that all possible genome structures are modeled in the data (from available data or through simulation) as are the variations in coverage, read lengths and error rates. The only long range information included has come from mate-pairs. Note, however, that our analysis is completely general, as is the software used in this study, and can be used for broader studies in the future.

For these reasons, following datasets were selected (see Table 2):

1. Sanger reads data, although now considered archaic, remain an important benchmark for the future. For instance, various technologies, promised by PacBiosciences, Life Technologies, and others, seek to match and exceed the read-lengths and accuracy of Sanger (hopefully, also inexpensively). Also Sanger-approach remains a statistically reliable source of data and implementations, since there continue to exist an active community of Sanger sequencers, a large amount of data and a variety of algorithmic frameworks dealing with Sanger data. As a result, they provide much more reliable statistics in the context of comparing all different algorithmic frameworks (e.g., greedy, OLC and SBH). Such richness is not yet available from the current short-read assemblers, which have primarily focused on SBH (and de Bruijn graph representation).
2. Most recent Illumina machines can now generate reads of about 100 bps or more, however, our focus on 36 bps Illumina reads is based on the fact the these datasets have been extensively analyzed by previously published short read assemblers. Since longer reads can only make the assembly process easier, these datasets still represent some of the hardest instances of the sequence assembly problem. We have also discovered that longer reads (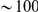 bps) from recent Illumina machines have higher error rates towards the ends of the reads, thus, limiting the apparent advantage of longer sequences.
3. By focusing on low-coverage long reads and short reads with high coverage, we wished to stress-test all assemblers against the most extreme instances of the sequence assembly problem, especially, where assembly quality is of essence.

**Table 2:** Benchmark data.

Genome	Length (bp)	Num. of reads	Avg. read length (bp)	Std. (bp)	Coverage
*Brucella suus*	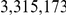	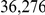	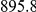	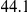	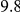
*Wolbachia sp.*	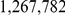	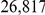	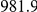	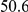	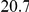
*Staphylococcus epidermidis*	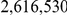	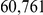	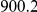	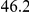	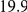
*Chromosome Y* 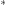	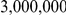	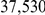	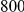	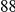	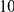
*Staphylococcus aureus*	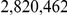	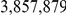	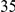	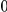	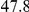
*Helicobacter acinonychis*	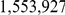	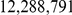	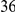	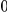	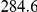
*Escherichia coli*	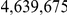	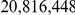	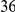	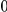	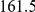

First and second columns report the genome name and length; columns 3 to 6 report the statistics of the shotgun projects: number of reads, average and standard deviation of the read length and genome coverage (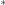
region [35,000,001–38,000,000]).

### Long reads

Starting with the pioneering DNA sequencing work of Frederick Sanger in 1975, every large-scale sequencing project has been organized around reads generated using the Sanger chemistry [28]. This technology could be typically characterized by reads of length up to 1000 bps and average coverage of 10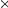. Additional mate-pair constraints are typically available in the form of estimated distance between a pair of reads.

The first data set of Sanger reads consists of three bacterial genomes: *Brucella suis* [29], *Wolbachia sp.* [30] and *Staphylococcus epidermidis* RP62A [31]. These bacteria have been sequenced and fully finished at TIGR, and all the sequencing reads generated for these projects are publicly available at two sites: the NCBI Trace Archive, and the CBCB website (www.cbcb.umd.edu/research/benchmark.shtml). Also included in the benchmark are sequence data from the human genome. Specifically, we selected a region of 3 Mb from human *Chromosome Y*'s p11.2 region. These euchromatin regions of Y Chromosome are assumed to be particularly challenging for shotgun assembly as it is full of pathologically complex patterning of genome structures at multiple scales and resolutions – usually described as fractal-like motifs within motifs (repeats, duplications, indels, head-to-head copies, etc.). For this region of the Y Chromosome we generated simulated shotgun reads as described in Table 2. We created two mate-pair libraries of size (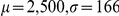) and (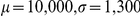) respectively; 90% of the reads have mates (45% from the first library and 45% from the second library), the rest of the reads are unmated; finally, we introduced errors in each read at a rate of 1%.

### Short reads

More recent advances in sequencing technology have produced a new class of massively parallel next-generation sequencing platforms such as: Illumina, Inc. Genome Analyzer, Applied Biosystems SOLiD System, and 454 Life Sciences (Roche) GS FLX. Although they have orders of magnitude higher throughput per single run (up to 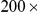 coverage) than older Sanger technology, the reads produced by these machines are typically shorter (35–500 bps). As a result they have introduced a succession of new computational challenges, for instance, the need to assemble millions of reads even for bacterial genomes.

For the short reads technology, we used three different data sets, which have been extensively analyzed by previously published short read assemblers. The first data set consists of 3.86 million 35-bp unmated reads from the *Staphylococcus aureus* strain MW2 [32]. The set of reads for this genome are freely available from the Edena assembler website (www.genomic.ch/edena.php). The second dataset consists of 12.3 million 36-bp unmated reads for a raw coverage of 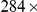. This second dataset is for the *Helicobacter acinonychis* strain Sheeba genome [33], which was presented in the SHARCGS [21] paper and is available for download at sharcgs.molgen.mpg.de. The third data set instead is made up of 20.8 million paired-end 36 bp Illumina reads from a 200 bp insert *Escherichia coli* strain K12 MG1655 [34] library (NCBI Short Read Archive, accession no. SRX000429).

### Assemblers

The following assemblers have been selected for comparison of long read pipelines: ARACHNE [14], CABOG [13], Euler [17], Minimus [15], PCAP [8], Phrap [6], SUTTA [25], and TIGR [5]. Similarly, the following assemblers were selected for comparison of short read pipelines: ABySS [19], Edena [16], Euler-SR [35], SOAPdenovo [20], SSAKE [22], SUTTA [25], Taipan [24], and Velvet [18]. Note that, although the most recent release of CABOG supports short reads from Illumina technology, it cannot be run on reads shorter than 64 bp.

This list is meant to be representative (see [36] for a survey), as it includes assemblers satisfying the following two criteria: (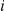) they have been used in large sequencing projects with some success, (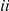) together they represent all the generally accepted assembly paradigms (e.g., greedy, OLC, SBH and B&B; see the discussion in the introduction) and (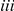) the source code or binaries for these assemblers are publicly available on-line, thus enabling one to download and run each of them on the benchmark genomes. In order to interpret the variability in assembler performance under different scenarios, both paired and unpaired data were analyzed separately. All the long-read assemblers were run with their default parameters, while parameters for the short-read assemblers were optimized according to recent studies [25] (see Table S1).

## Results

In order to analyze the assembly quality of many different assemblers and to understand the inconsistency of many traditional metrics, it was decided to collect a significant volume of comparative performance statistics using a large benchmark of both bacterial and human genome data. For all the genomes used in this study, the finished sequences are available, thus enabling direct validation of the assemblies.

### Long reads results

#### Analysis without mate-pair constraints

Table 3 presents the contig size analysis while excluding mate-pair data (thus, ignoring clone sizes and forward-reverse constraints). Since not all next-generation sequencing technologies are likely to produce mate-pair data, it is informative to calibrate to what extent an assembler's performance is determined by such auxiliary information. Note that ARACHNE had to be omitted from this comparison (and the associated Table 3), since its use of mate-pairs is tightly integrated into its assembly process and cannot be decoupled from it. Similarly CABOG does not support data that is totally lacking in paired ends.

**Table 3:** Long reads comparison (without mate-pairs).

Genome	Assembler	# contigs	# big contigs ( 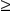 10 kbp)	Max (kbp)	Mean big contigs (kbp)	N50 (kbp)	Big contigs coverage (%)
*Brucella*	Euler	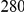	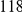	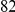	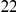	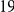	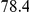
*suis*	Minimus	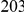	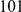	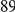	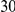	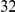	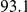
	PCAP	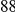	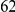	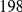	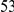	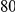	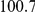
	PHRAP	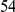	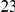	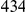	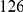	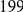	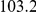
	SUTTA	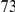	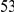	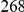	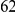	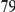	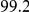
	TIGR	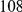	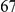	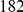	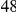	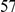	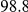
*Staphylococcus*	Euler	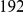	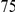	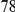	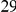	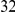	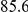
*epidermidis*	Minimus	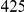	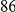	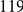	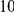	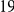	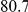
	PCAP	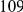	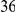	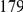	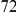	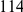	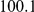
	PHRAP	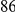	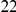	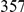	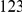	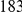	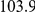
	SUTTA	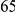	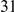	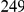	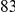	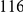	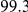
	TIGR	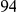	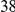	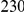	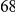	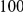	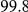
*Wolbachia sp.*	Euler	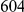	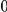	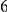	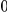	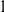	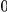
	Minimus	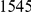	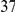	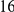	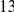	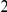	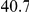
	PCAP	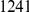	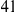	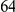	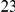	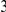	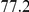
	PHRAP	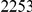	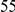	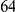	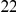	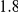	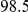
	SUTTA	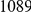	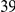	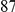	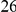	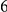	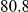
	TIGR	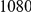	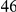	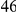	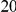	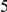	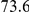
*Human*	Euler	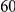	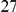	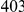	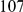	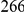	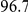
*Chromosome Y*	Minimus	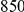	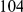	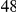	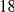	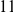	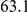
	PCAP	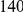	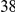	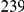	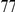	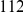	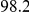
	PHRAP	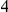	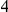	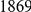	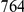	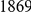	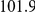
	SUTTA	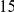	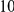	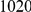	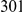	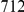	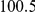
	TIGR	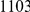	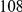	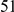	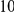	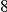	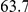

Long reads assembly comparison without mate-pair information (clone sizes and forward-reverse constraints). First and second columns report the genome and assembler name; columns 3 to 7 report the contig size statistics, specifically: number of contigs, number of contigs with size 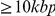, max contig size, mean contig size, and N50 size (N50 is the largest number 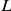 such that the combined length of all contigs of length 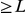 is at least 50% of the total length of all contigs). Finally column 8 reports the coverage achieved by the large contigs (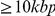). Coverage is computed by double-counting overlapping regions of the contigs, when aligned to the genome.

A caveat with the preceding analysis needs to be addressed: if, one wishing to select an assembler of the highest quality, were to base one's judgement solely on the standard and popular metrics (as in this Table 3), the result would be somewhat uncanny and unsatisfying. For instance, Pharp would appear to be a particularly good choice, since it seems to typically produce an almost complete genome coverage with fewer contigs, but each of sizable lengths (as confirmed by the N50 values). More specifically, except for *Wolbachia*, the N50 value of Phrap is the highest, yielding a respectable genome coverage by the big contigs (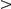
10 kbp). Unfortunately, a closer scrutiny of the Phrap-generated assembly (e.g., the dot plots of the contigs' alignment) reveals that Phrap's apparent superiority is without much substance – Phrap's weaknesses, as evidenced by its mis-assemblies within long contigs (see alignments in Document S1), are not captured by the N50-like performance parameters. Phrap's greedy strategy cannot always handle long-range genome structures and when a repeat boundary is found it can be fooled by false positive overlaps. In contrast TIGR, PCAP and SUTTA have similar performance in terms of N50; however, SUTTA produces a smaller number of big contigs (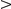
10 kbp) compared to TIGR and PCAP, and higher genome coverage (except for the *S. epidermidis*, where they have similar coverage). All the assemblers encounter various difficulties in assembling the *Wolbachia sp.* dataset into long contigs, which is probably due to a higher error rate in the reads. These difficulties are conspicuously noticeable for Euler assembler; in fact, its big contigs coverage comes very close to zero. Minimus instead uses a very conservative approach where, if a repeat boundary is encountered, it stops extending the contig. Such strategy reduces the possible mis-assembly errors, but pays a considerable price in contig size.

The results for the 3 Mb region of *Chromosome Y* (from p11.2, a euchromatin region) instead paint a somewhat different picture. TIGR's and PCAP's performances are now inferior, with lower coverage and higher number of contigs generated. In particular PCAP performance was obtained by reducing the stringency in overlap detection to tolerate more overlaps (using parameter -d 500), this parameter setting was necessary in order to generate reasonably long contigs. Phrap still has the best performances in terms of contig size and N50, followed by SUTTA, but now its alignment results do not show mis-assembled contigs (see dot plots in Document S1). Also, surprisingly Euler now improves the genome coverage for simulated assembly. Note that for *Chromosome Y* simulated reads were generated using fairly realistic error distributions, but still raise questions about the simulation's fidelity (e.g., ability to capture the non uniform coverage pattern, potential cloning bias, etc., that would be inevitable in any real large scale genomic project). Various simplifying assumptions used by the simulators may explain why simulated data appear somewhat easier to assemble.

**Figure 1:**
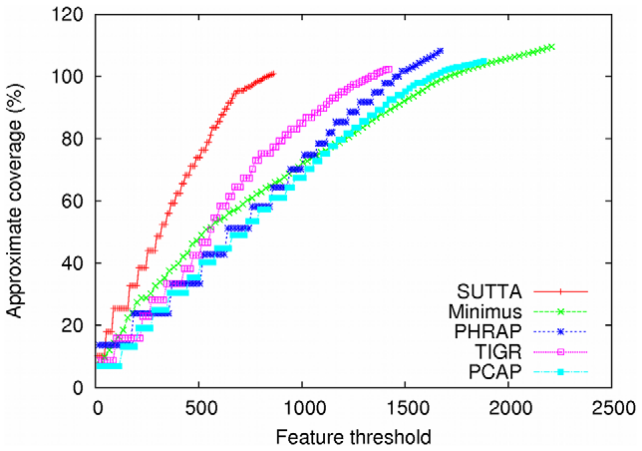
Feature-Response curve comparison for *S. epidermidis*. For this comparison no mate-pairs information was used in the assembly.

**Figure 2:**
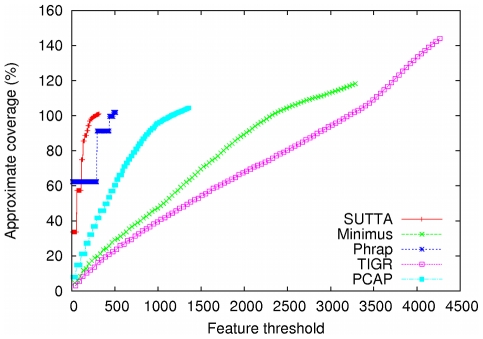
Feature-Response curve comparison for *Chromosome Y* (3 Mbp of p11.2 region). For this comparison no mate-pairs information was used in the assembly.

Since the contig size analysis gives only an incomplete and often misleading view of the real performance of the assemblers, a more principled and informative approach needs to be devised. As described earlier, with this in mind, a new metric, called “Feature-Response curve” (FRC), is proposed and evaluated to see how well it can check the quality of the contigs and validate the assembly output. Figures 1 and 2 show the Feature-Response curve comparison for the *S. epidermidis* and *Chromosome Y* (p11.2 region) genomes when mate-pairs are not used in the assembly. The 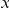-axis is the maximum number 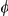 of errors/features allowed in the contigs and the 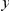-axis reports the approximate genome coverage achieved by all the contigs (sorted in decreasing order by size) such that the sum of their features is 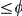 (see Methods for more details). Note that the definition of coverage used in this plot is not the conventional one since we double-count overlapping regions of contigs, when aligned to the genome. We decided to employ such a definition because it highlights assemblies that over-estimate the genome size (coverage greater than 100%). Based on this analysis, SUTTA seems to be performing better than all the other assemblers in terms of assembly quality, however it is important to mention that the current version of the FRC includes several types of assembly errors with a uniform weighting, chosen arbitrarily. For example, a mis-join is generally considered the most severe type of mis-assembly, but this is not currently captured by the FRC. In fact SUTTA clearly creates mis-joined contigs in the absence of paired reads (see dot plots in Document S1), however this problem is alleviated by the addition of paired reads as shown next in the analysis with mate-pair constraints. Finally note that Euler and Minimus go to extreme lengths to avoid mis-joins in the absence of paired reads.

#### Analysis with mate-pair constraints

Table 4 presents the results with mate-pairs data, restricting the analysis only to assemblers (ARACHNE, CABOG, Euler, PCAP, SUTTA and TIGR) that use mate-pair constraints effectively during the assembly process. Obviously, the use of mate-pair-constraints improves the performance and quality of all four assemblers; however, they do so to varying degrees. For example TIGR's N50 values are now typically twice as large as those without mate-pairs. In contrast, Euler's results only improve marginally with mate-pair constraints, and it is still unable to produce contigs larger than 10 kbp for the *Wolbachia sp.* genome. Note that Euler shows weaker performance in comparison to the results reported on its home-page (http://nbcr.sdsc.edu/euler/benchmarking/bact.html) for the bacterial genomes. Although the exact explanation of this discrepancy is not obvious, it could be due to an additional screening (preprocessing) of the reads that removes low quality regions (note that, here, the analysis of all assemblers assumes no preprocessing.). ARACHNE and CABOG exhibit the highest N50 values for all datasets.

**Table 4:** Long reads comparison (with mate-pairs).

Genome	Assembler	# contigs	# big contigs ( 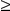 10 kbp)	Max (kbp)	Mean big contigs (kbp)	N50 (kbp)	Big contigs coverage (%)
*Brucella*	ARACHNE	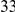	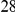	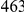	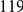	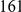	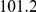
*suis*	CABOG	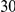	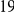	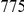	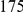	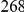	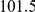
	Euler	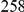	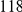	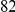	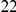	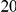	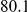
	PCAP	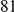	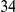	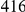	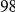	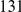	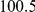
	SUTTA	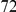	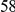	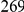	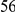	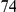	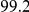
	TIGR	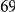	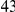	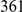	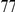	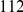	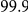
*Staphylococcus*	ARACHNE	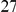	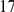	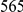	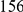	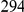	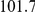
*epidermidis*	CABOG	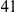	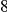	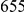	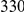	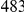	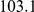
	Euler	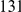	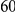	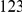	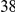	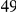	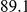
	PCAP	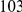	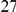	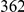	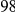	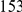	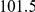
	SUTTA	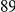	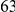	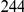	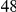	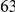	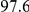
	TIGR	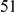	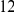	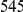	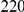	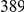	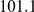
*Wolbachia sp.*	ARACHNE	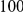	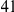	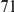	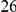	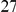	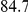
	CABOG	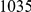	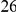	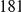	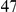	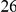	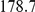
	Euler	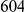	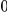	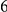	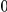	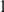	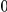
	PCAP	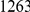	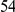	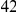	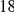	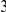	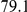
	SUTTA	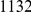	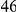	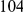	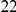	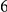	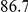
	TIGR	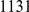	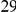	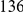	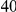	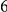	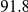
*Human*	ARACHNE	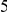	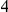	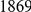	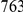	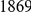	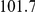
*Chromosome Y*	CABOG	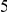	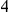	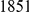	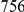	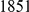	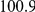
	Euler	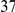	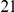	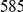	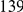	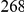	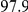
	PCAP	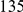	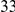	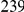	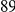	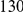	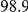
	SUTTA	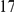	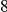	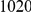	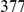	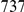	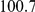
	TIGR	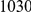	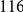	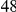	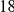	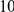	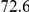

Long reads assembly comparison using mate-pair information. First and second columns report the genome and assembler name; columns 3 to 7 report the contig size statistics, specifically: number of contigs, number of contigs with size 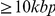, max contig size, mean contig size, and N50 size (N50 is the largest number 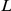 such that the combined length of all contigs of length 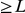 is at least 50% of the total length of all contigs). Finally column 8 reports the coverage achieved by the large contigs (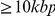). Coverage is computed by double-counting overlapping regions of the contigs, when aligned to the genome.

**Figure 3:**
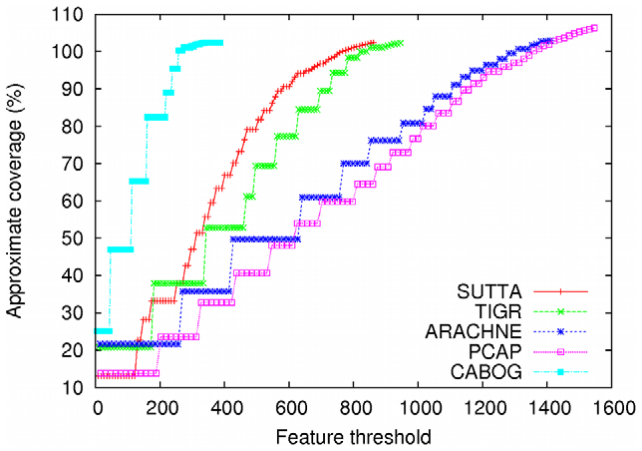
Feature-Response curve comparison for *S. epidermidis*. For this comparison mate-pairs information was used in the assembly.

**Figure 4:**
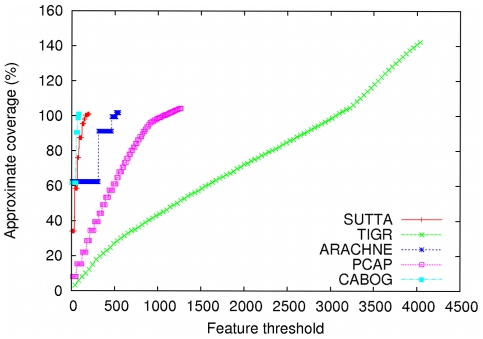
Feature-Response curve comparison for *Chromosome Y* (3 Mbp of p11.2 region). For this comparison mate-pairs information was used in the assembly.

**Figure 5:**
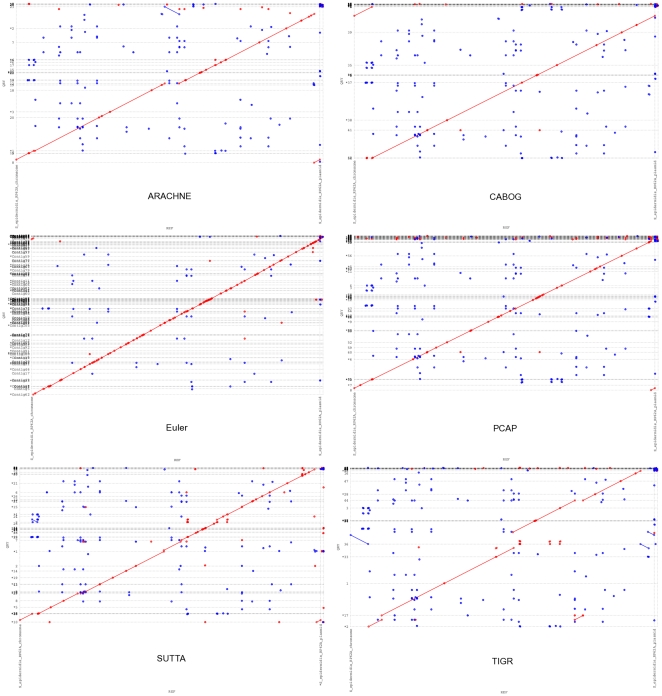
Dot plots of the assemblies for *Staphylococcus epidermidis*. Assemblies generated by ARACHNE, CABOG, Euler, PCAP, SUTTA and TIGR. The horizontal lines indicate the boundary between assembled contigs represented on the 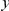 axis. Note that number of single dots are an artifact of the sensitivity of the MUMmer alignment tool; they can be reduced or removed using a larger value for the minimum cluster length parameter –*mincluster* (default 65).

As earlier, while the contig size analysis indicates all of the following assemblers, ARACHNE, CABOG, PCAP and TIGR producing better performance, a cursory inspection of the Feature-Response curve points to a different conclusion. Figures 3 and 4 show the Feature-Response curve comparison for *S. epidermidis* and *Chromosome Y* (p11.2 region) genomes when mate-pairs are used in the assembly (note: because Euler assembly output could not be converted into an AMOS bank for validation, it is excluded from this plot). An intuitive understanding of the different assembly quality can be gleaned from Figure 5 which shows the dot plots of comparison of assemblies produced by the various assemblers, aligned to the completed *S. epidermidis* genome. The dot plot alignments were generated using the MUMmer package [37] (http://mummer.sourceforge.net/). Assemblies generated by CABOG, Euler, PCAP and SUTTA are seen to match quite well with the reference sequence, as suggested by the fraction of matches lying along the main diagonal. Note that since *S. epidermidis* has a circular genome, the small contigs aligned at the bottom right or top left are not mis-assembled. TIGR instead shows many large assembly errors, mostly due to chimeric joining of segments from two distinct non-adjacent regions of the genome. Further, note that two perfect dot plot alignments can still have different quality when analyzed with the FRC. An example is given by comparing the contigs generated by ARACHNE and SUTTA for the 3 Mb segment of *Chromosome Y*'s p11.2. Despite the dot plots showing high alignment quality for both, the FRC scores them very differently (see FRCs in Document S1). Document S1 contains the dot plots for the other genomes and the associated FRCs.

**Figure 6:**
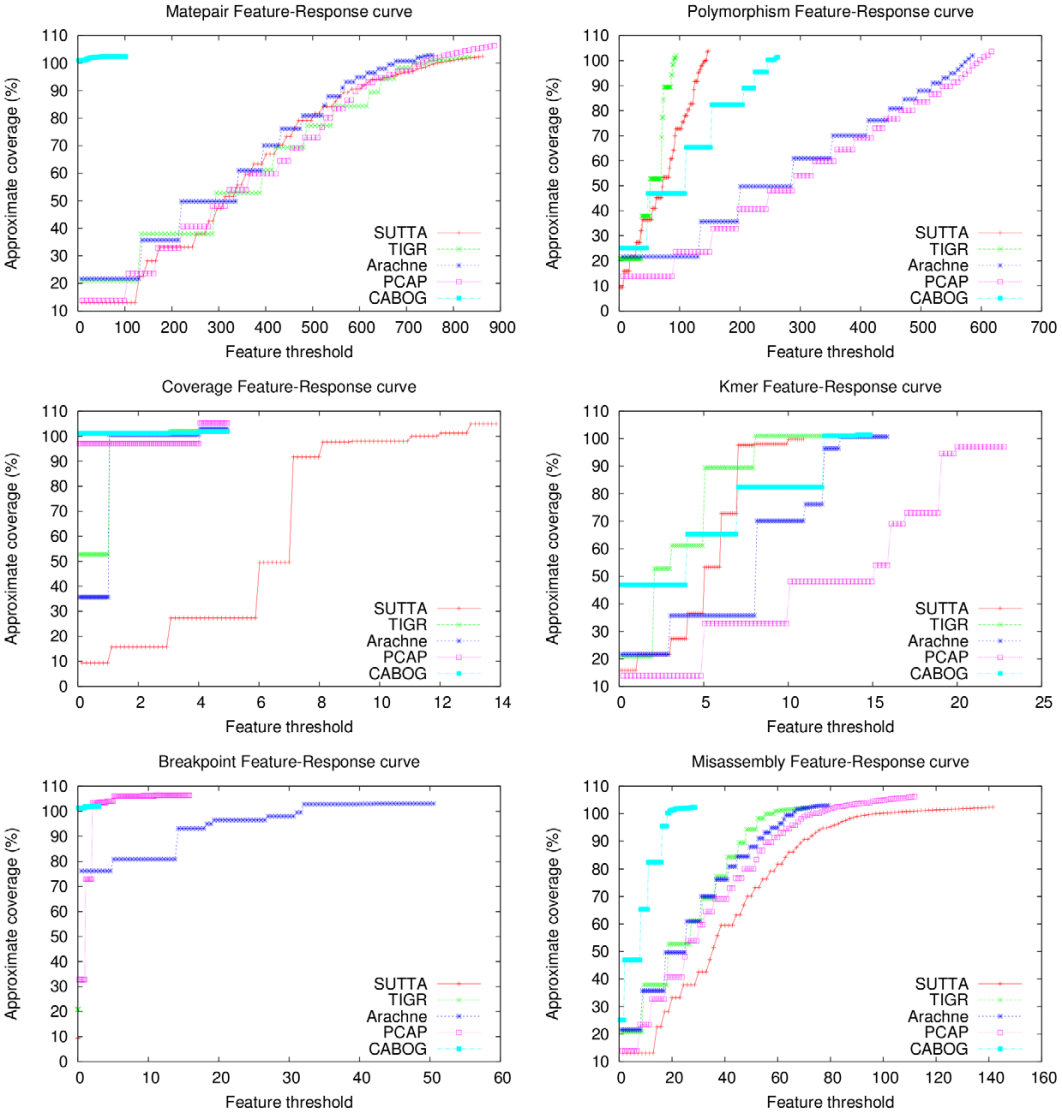
Feature-Response curve comparison by feature type for *S. epidermidis* using mate-pairs.

To further analyze the relative strengths and weaknesses of each assembler, Figure 6 shows separate FRCs for each feature type when assembling the *S. epidermidis* genome using mate-pairs. By inspecting these plots it is clear that each assembler behaves differently according to each feature types. For example CABOG outperforms the other assemblers when mate-pair constraints are considered. TIGR and SUTTA outperform the other assemblers in the number of correlated polymorphism in the read alignments. While the FRC that analyzes the depth of coverage, shows ARACHNE, CABOG and PCAP to be winners in the comparison. Moving to the FRC that analyzes the 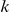-mer frequencies, which can be used to detect the presence of mis-assemblies due to repeats, SUTTA and TIGR outperform ARACHNE and PCAP, while CABOG performs somewhere in between. The breakpoint-FRC examines the presence of multiple reads that share a common breakpoint, which often indicate assembly problems. PCAP and ARACHNE seem to suffer more from this problems, while the other assemblers are not affected (the FRCs reduces to a single point). Finally the mis-assembly FRC is computed using the mis-assembly feature which is obtained applying a feature combiner to collect a diverse set of evidence for a mis-assembly and output regions with multiple mis-assembly features present at the same region (see [38] for more details). CABOG in this case returns to a superior rank over the other assemblers.

### Short reads results

In case of short reads, interpretations of the contigs data, e.g., ones based purely on contig sizes and N50, etc. are complicated by the following facts: (1) for short-reads, the required threshold ratio 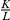 is only slightly less than 1, where 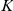 is the required minimum overlap length and 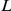 is the length of the reads; therefore the effective coverage is significantly small, thus making all the statistics rather non-robust and highly sensitive to choice of the parameters; (2) there is no consensus definition of correctness of a contig – the required similarity varying from 98% down to 90% and the allowed end-trimming of each contigs being idiosyncratic; and (3) many algorithms have specific error-correction routines that are embedded in a pre-processing or post-processing steps and that cull or correct bad reads and contigs in a highly technology-specific manner.

#### Analysis without mate-pair constraints

Returning to an analysis based on contig size, in Table 5, we show a comparison of the assembly results for the *S. aureus* and *H. acinonychis* genomes. The values reported for all the assemblers, are based on the tables presented in the recent SUTTA paper [25]. Only contigs of size 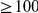 are used in the statistics. Without mate-pair information, as in here, it is inevitable that all assembly approaches (especially, if they are not conservative enough) could produce some mis-assemblies. As described earlier, what constitutes a correct contig is defined idiosyncratically (align along the whole length with at least 98% base similarity [16]), making it very sensitive to small errors which typically occur in short distal regions of the contigs. For example, contigs ending in gaps accumulate errors, as coverage gets lower towards the ends. To overcome such errors some assemblers perform a few correction steps. For example, Edena exercises an option to trim a few bases from these ends until a minimum coverage is reached; Euler-SR performs a preprocessing error correction step where errors in reads are corrected based on 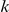-mer coverage analysis. From the Table 5 it is clear that SSAKE has the worst performance in terms of contig size and quality, while the rest of the assemblers have relatively small errors and they all achieve high genome coverage (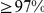).

**Table 5:** Short reads comparison (without mate-pairs).

Genome	Assembler	# correct	# mis-assembled	N50 (kbp)	Mean (kbp)	Max (kbp)	Coverage (%)
*S. aureus*	ABySS	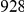	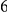	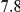	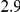	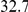	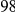
(strain MW2)	Edena (strict)	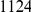	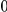	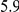	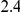	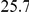	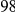
	Edena (nonstrict)	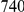	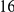	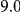	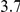	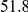	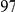
	EULER-SR	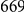	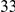	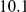	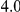	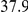	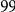
	SOAPdenovo	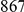	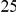	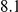	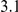	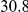	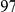
	SSAKE	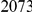	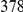	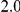	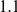	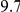	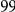
	SUTTA	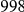	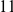	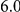	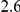	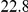	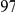
	Taipan	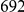	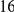	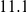	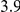	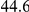	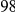
	Velvet	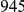	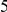	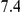	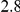	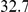	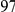
*H. acininychis*	ABySS	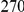	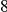	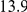	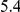	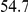	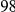
(strain Sheeba)	Edena (strict)	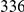	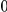	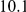	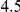	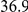	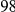
	Edena (nonstrict)	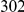	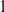	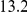	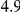	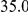	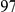
	EULER-SR	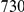	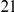	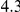	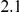	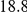	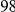
	SOAPdenovo	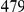	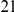	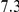	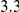	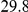	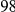
	SSAKE	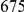	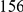	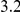	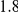	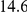	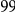
	SUTTA	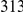	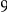	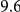	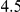	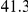	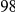
	Taipan	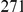	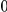	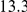	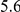	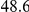	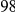
	Velvet	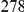	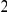	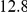	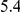	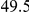	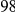

Short reads assembly comparison without mate-pair information. First and second columns report the genome and assembler name; columns 3 to 7 report the contig size statistics, specifically: number of contigs, number of contigs with size 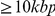, max contig size, mean contig size, and N50 size (N50 is the largest number 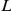 such that the combined length of all contigs of length 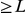 is at least 50% of the total length of all contigs). Finally column 8 reports the coverage achieved by all the contigs.

#### Analysis with mate-pair constraints

Table 6 shows the assembly comparison using mate-pair information on the read set for the *E. coli* genome. The comparison is based on the results from Table 2 in [25]. In accordance with this analysis, statistics are computed only for contigs whose length is greater than 100 bps. A contig is defined to be correct if it aligns to the reference genome with fewer than five consecutive base mismatches at the termini and has at least 95% base similarity. By inspecting the column with the number of errors, one might conclude that lower is the number of errors better is the overall assembly quality. As explained earlier, a simple count of the number of total mis-assembled contigs is not informative enough. For example ABySS and SOAPdenovo have the highest N50 values and low number of mis-assembled contigs, however such mis-assembled contigs are on average longer than those from other assemblers like SUTTA and Edena. This is evident in the table from the analysis of the mean length of the mis-assembled contigs. This analysis also shows that Edena and SUTTA behave more conservatively than Velvet, ABySS and SOAPdenovo, as they trade contig length in favor of shorter correctly assembled contigs. Interestingly Taipan's number of errors for *E .coli* increases compared to the results in Table 5. Instead SOAPdenovo's performance improves for *E.coli* thanks to the availability of mate-pair information.

**Table 6:** Short reads comparison (with mate-pairs).

Genome	Assembler	# correct	# mis-assembled (mean kbp)	N50 (kbp)	Mean (kbp)	Max (kbp)	Coverage (%)
*E. coli*	ABySS	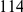	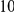 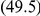	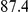	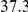	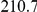	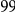
(K12 MG1655)	Edena	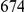	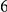 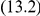	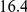	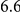	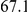	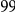
	EULER-SR	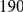	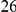 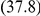	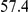	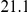	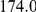	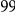
	SOAPdenovo	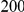	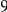 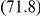	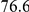	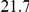	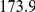	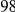
	SSAKE	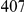	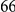 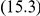	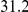	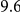	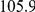	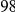
	SUTTA	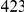	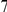 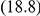	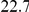	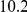	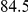	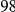
	Taipan	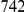	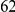 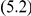	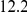	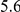	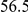	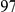
	Velvet	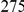	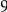 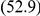	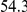	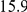	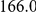	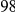

Short reads assembly comparison using mate-pair information. First and second columns report the genome and assembler name; columns 3 to 7 report the contig size statistics, specifically: number of contigs, number of contigs with size 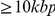, max contig size, mean contig size, and N50 size (N50 is the largest number 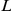 such that the combined length of all contigs of length 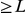 is at least 50% of the total length of all contigs). Finally column 8 reports the coverage achieved by all the contigs.

**Figure 7:**
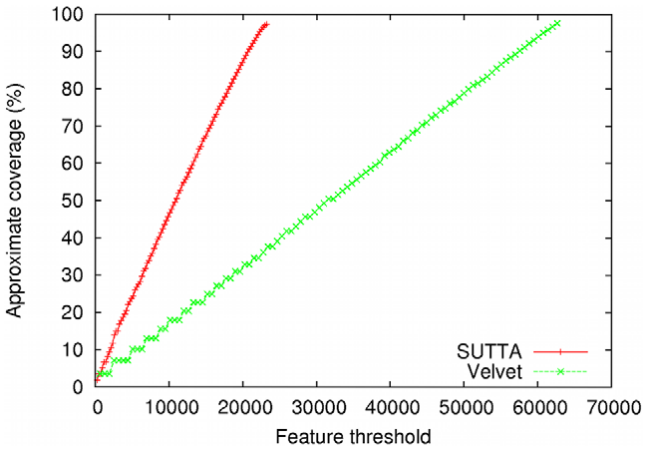
Feature-Response curve comparison for *E. coli*. For this comparison mate-pairs information was used in the assembly.

Note that the N50 statistic does not give any information about the reason why the contigs are mis-assembled: the contigs could contain an error due to accumulated errors close to the contigs' ends or it could contain rearrangements due to repeated sequences. Of course the two error types have very different contributions and roles in terms of quality. In this context the FRC analysis can give a deeper understanding of the assembly quality, as shown in Figure 7 where the contigs produced by SUTTA and Velvet are compared. Although SUTTA has a higher number of mis-assembled contigs (see Table 6), the FRC presents a different scenario. By inspecting the feature information of the contigs produced by SUTTA and Velvet, it is seen that SUTTA's contigs have lower number of unsatisfied mate-pair constraints, which leads to fewer large mis-assembly errors. This result is primarily due to SUTTA's optimization scheme, which allows it to concurrently optimize both overlap and mate-pair scores while searching for the best layout. Unfortunately we were unable to generate FRCs for each short-read assembler because their output could not be converted into an AMOS bank for validation. The ones that could be analyzed with FRC include SUTTA and Velvet.

## Discussion

A recent article, entitled ÒRevolution PostponedÓ in Scientific American [39] described, “The Human Genome Project has failed so far to produce the medical miracles that scientists promised. Biologists are now divided over what, if anything, went wrong…”. And yet, the excitement over the rapid improvements in biochemistry (pyrosequencing, sequencing by synthesis, etc.) and sensing (zeroth-order waveguides, nanopores, etc.) has now pervaded the field, as newer and newer sequencing platforms have started mass-migration into laboratories. Thus, biologists stand at a cross-road, pondering over the question of how to tackle the challenges of large-scale genomics with the high-throughput next-gen sequencing platforms. One may ask: Do biologists possess correct reference sequence(s)? If not, how should they be improved? How important are haplotypes? Does it suffice to impute the haplotype-phasing from population? How much information is captured by the known genetic variants (e.g., SNPs and CNVs)? How does one find the de novo mutations and their effects on various complex traits? Can exon-sequencing be sufficiently informative?

Central to all these challenges is the one problem we have addressed in this paper: namely, how correct are the existing sequence assemblers for making reference genome sequences? How good are the assumptions they are built upon? Unfortunately we have discovered that the quality and performance of the existing assemblers vary dramatically. In addition standard metrics used to compare assemblers for the last ten years emphasize contig size while poorly capturing the assembly quality. For these reasons we have developed a new metric, Feature-Response curve (FRC), to compare assembles that more satisfactory captures the trade-off between contigs' size and quality. This metric shares many similarities with the receiver operating characteristic curve widely used in medicine, radiology, machine learning and other areas for many decades. Also the FRC does not require any reference sequence (except an estimate of the genome size) to be used for validation, thus making it a very useful tool in *de novo* sequencing projects. Furthermore the inspection of separate FRCs for each feature type enables to scrutinize the relative strengths and weaknesses of each assembler. The current formulation of the FRC is exceedingly simple and yet natural. Thus, we hope that starting from here more sophisticated versions of the FRC will be developed in the future. For example, the features could be weighted by contig length (density function); additional features may be included; features may be combined or transformed (e.g., eigen-features); the response, instead of coverage, could be another assembly quality metric of choice; etc. It must be emphasized that the features should not be interpreted directly as errors, since, as reported by the developers of *amosvalidate* [38], the method used to compute each feature may contain some false-positives. These false-positives frequently correspond to irresolvable inconsistencies in the assembly – and not mis-assembly errors or incorrect consensus sequence. Consequently, the results could appear pessimistic for any one assembler, but are unlikely to be skewed in a comparative study, such as the one here. The utility of Feature-Response curve is thus not diminished by the nature of the simple features, and it should be used in combination with other metrics and alignments to the reference genome (if available). Note further that since the reported sensitivity of *amosvalidate* is 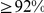, almost all the mis-assemblies are captured by one or more features, pointing to possible sources of errors in a particular assembler.

Note that although our aim has been to test and compare as many *de novo* sequence assemblers and covering known assembly paradigms as exhaustively as possible, in a fast evolving field such as this, this goal has not been completely met – some of the assemblers listed in Table 1 were only released very recently, not early enough to be included in the statistical comparison. It is hoped that the community of researchers interested in sequence assembly algorithms will close this gap using the FRC software, now available as part of the AMOS open-source consortium. No doubt, our team will also publish incremental updates on our website as we complete further statistical analysis.

Returning to our earlier concerns and mirroring quandaries of computational biologists, biotechnologists also need to reflect on related issues: Given the unavoidable computational complexity burden of assembly, how best to design the sequencing platforms? There are multiple parameters that characterize a sequencing platform: read-length, base-call-errors, homopolymer-length, throughput, cost, latency, augmentation with mate-pairs, scaffolding, long-range information, etc. And not all can be addressed equally well in all the platforms. For the time being, low-resolution map technology (e.g., optical restriction maps) for very long immobile molecules points to the most profitable avenue. If one were to speculate what the next step should be, as was done by Schwartz and Waterman [2], one may “project that over the next two years, reference genomes will be constructed using new algorithms combining long-range physical maps with voluminous Gen-2/3 datasets. In this regard, the Optical Mapping System constructs genome-wide ordered restriction maps from individual (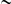
500 kbp) genomic DNA molecules. ” [25], [40]–[45] Among all the assemblers examined here, SUTTA appears to be best suited for such a strategy.

## Materials and Methods

### Feature-Response Curve

**Figure 8:**
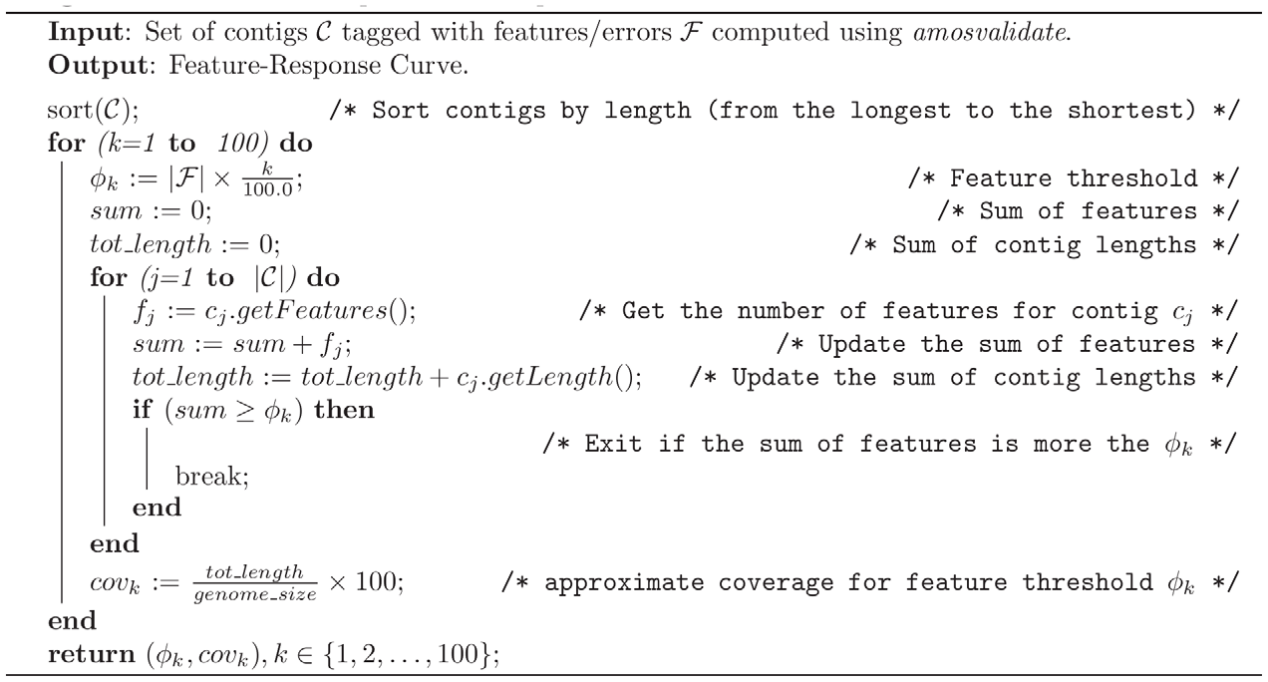
Algorithm pseudo-code for computing the Feature-Response curve.

Inspired by the standard receiver operating characteristic (ROC) curve, the Feature-Response curve characterizes the sensitivity (coverage) of the sequence assembler as a function of its discrimination threshold (number of features). The AMOS package provides an automated assembly validation pipeline called *amosvalidate* [38] that analyzes the output of an assembler using a variety of assembly quality metrics (or features). Examples of features include: (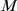) mate-pair orientations and separations, (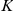) repeat content by k-mer analysis, (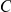) depth-of-coverage, (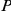) correlated polymorphism in the read alignments, and (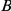) read alignment breakpoints to identify structurally suspicious regions of the assembly. After running amosvalidate on the output of the assembler, each contig is assigned a number of features that correspond to doubtful regions of the sequence. For example in the case of mate-pairs checking (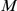) the tool flags regions where multiple matepairs are mis-oriented or the insert coverage is low. Given any such set of features, the response (quality) of the assembler output is then analyzed as a function of the maximum number of possible errors (features) allowed in the contigs. More specifically, for a fixed feature threshold 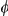, the contigs are sorted by size and, starting from the longest, only those contigs are tallied, if their sum of features is 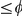. For this set of contigs, the corresponding approximate genome coverage is computed, leading to a single point of the Feature-Response curve. Figure 8 shows the algorithm pseudo-code for the FRC computation.

Note that no reference sequence is used to compute the FRC curve, which makes the FRC a useful tool in de novo sequencing project where a reference genome is not available to validate and guide the assembly process. In a scenario where the size of the genome is not available any reasonably good estimate of the reference genome size is adequate for the purpose of computing the FRC, since the genome size is simply used as a normalizing denominator across all the assemblers to compare the contigs quality. For instance, in the case of re-sequencing, a good estimate for the genome size can be obtained from genomes of the related species. In the case of the de novo sequencing projects the genome size can be judged from estimate of coverage (usually modeled as a dispersed Poisson) from a subsample of contigs (with some care for eliminating the outliers coming from repeats or difficult to sequence regions). The procedure described in Figure 8 can be applied to generate FRCs for each feature separately as shown in Figure 6. Finally note that the definition of coverage computed by the FRC is only an approximation of the standard one because the contigs are not aligned to the genome, however it has the property of identifying assemblies where the genome length has been over estimated.

### Implementation details

The Feature-Response curve has been developed as part of the AMOS assembly framework (A Modular Open-Source assembler, http://amos.sourceforge.net), a tool developed by a consortium of institutions and research centers associated with the University of Maryland. In order to facilitate the interaction of many isolated components, AMOS provides a central data repository of various genomic objects (reads, inserts, maps, overlaps, contigs, scaffolds, etc.) to be easily collected and indexed. In addition, the framework already provides several algorithms to perform some of the standard steps in the assembly pipeline (e.g., Trimming, Overlapping, Error Correction, Scaffolding, Validation). Following the AMOS philosophy, the FRC is implemented as a pipeline that consists of two steps: 1) invocation to the *amosvalidate* tool to compute the features for the set of contigs; 2) invocation to the FRC module that implements the algorithm illustrated in Figure 8.

## Supporting Information

Document S1 **Complete experimental results.** This document contains the full set of results on on the dat sets used in the paper, specifically: (1) separate Feature-Response curves for each feature type, (2) dot plots of the assembled contigs aligned to the reference genome.
(PDF)

Table S1 **Parameter setting used for each assembler.**
(PDF)
