# Supplementary material for: Comparing De Novo Genome Assembly: The Long and Short of It
Source: PLoS One. 2011 Apr 29;6(4):e19175. doi: 10.1371/journal.pone.0019175 (PMC3084767; doi:10.1371/journal.pone.0019175)
Supplement: Document S1 — Complete experimental results. This document contains the full set of results on on the dat sets used in the paper, specifically: (1) separate Feature-Response curves for each feature type, (2) dot plots of the assembled contigs aligned to the reference genome. (PDF) [file pone.0019175.s001.pdf]

Supporting information

The supplementary material S1 is organized in two main sections:

Long Reads

1. No Mate-Pairs constraints
   (a) *Brucella suis*
   (b) *Staphylococcus epidermidis*
   (c) *Wolbachia Sp.*
   (d) *Chromosome Y*
2. With Mate-Pairs constraints
   (a) *Brucella suis*
   (b) *Staphylococcus epidermidis*
   (c) *Wolbachia sp.*
   (d) *Chromosome Y*

Short Reads

1. With Mate-Pairs constraints
   (a) *Escherichia coli*

For each genome we report the Feature-Response curves (FRC) cumulative over all the features, the FRCs for each feature type, and the dot plot alignments of the set of contigs generated by each assembler computed using the MUMmer[1] package. For the short read E. coli data, only Velvet is used in the comparison since it is the only short read assembler whose output can be converted into an AMOS bank for validation. For the same reason Euler is excluded from the dot plots for long reads. The mis-assembly

[1] http://mummer.sourceforge.net/

FRC is computed using the mis-assembly feature which, according to the *amosvalidate* description, is obtained by applying a feature combiner that collects all of the evidence for a mis-assembly and outputs regions with multiple mis-assembly features when present at the same region. Note that the mis-assembly feature is not used in computing the FRC curve. Finally note that when the number of features of a specific type is 0 for each contig in the set, the FRC reduces to a single point. Although not all the curves are equally informative, for the sake of completeness of exposition, we present all the FRCs.

1 Long Reads

1.1 No Mate-Pairs constraints

Brucella suis

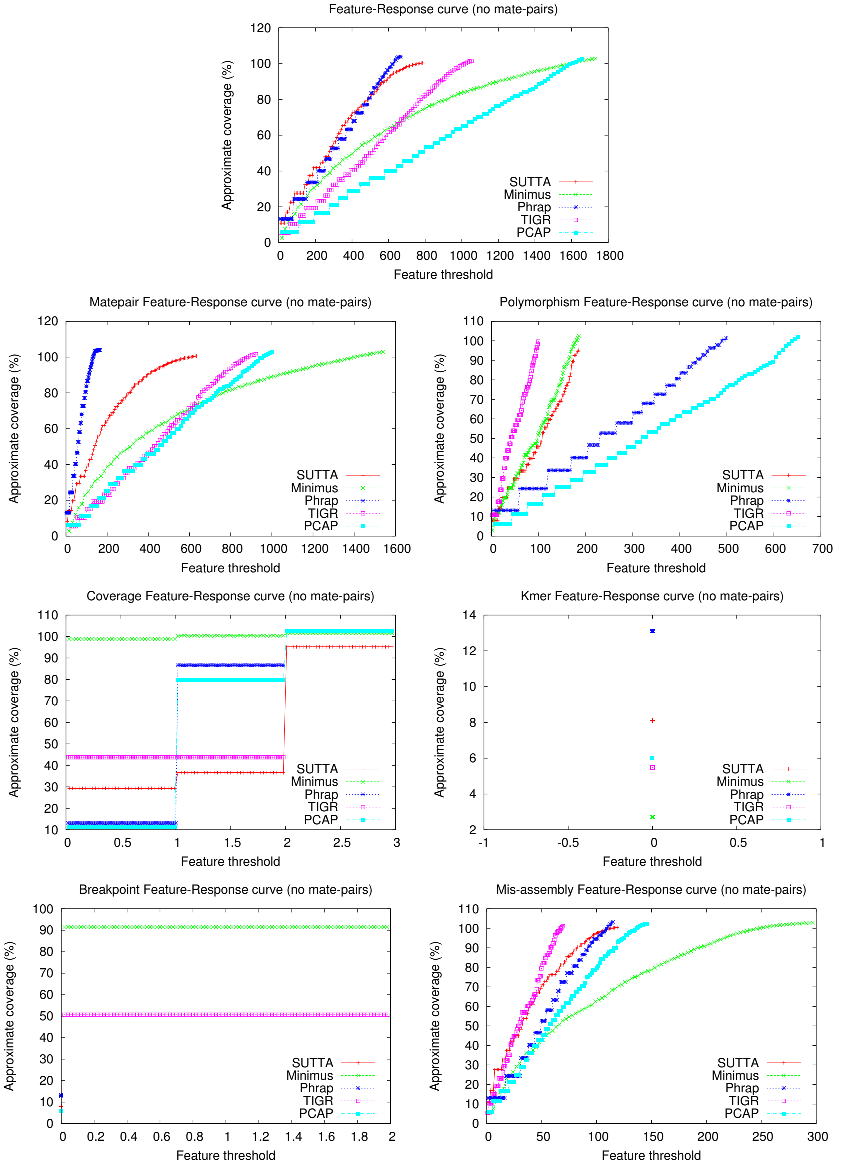

Figure 1: Feature-Response curves.

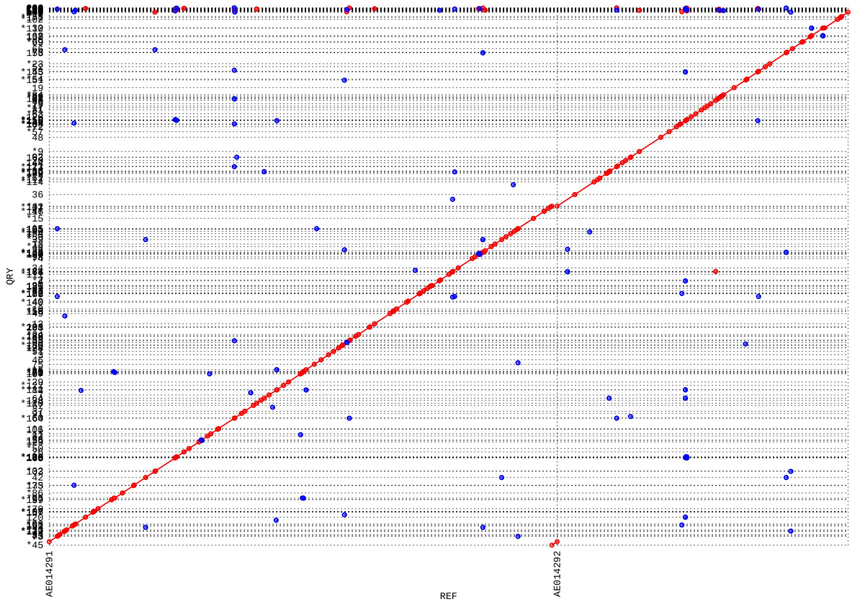

Figure 2: Minimus dotplot.

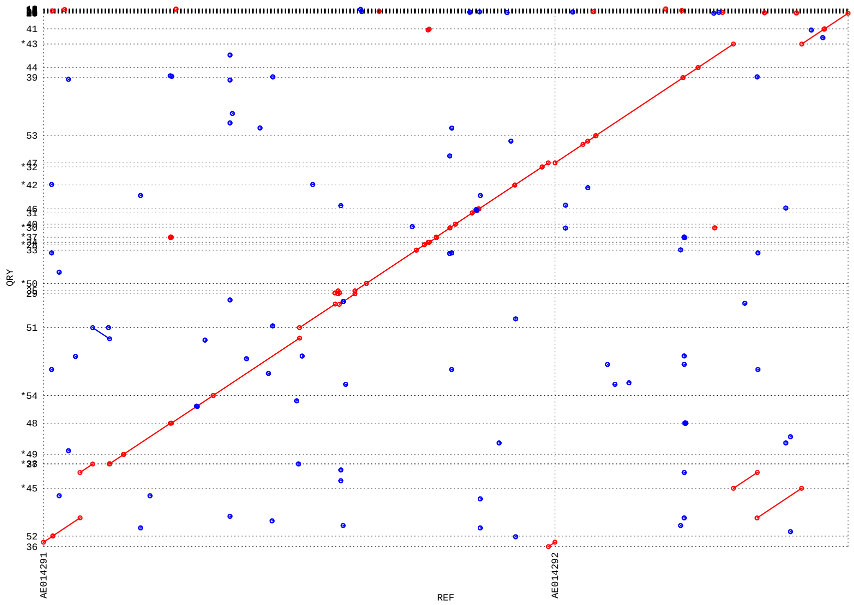

Figure 3: Phrap dotplot.

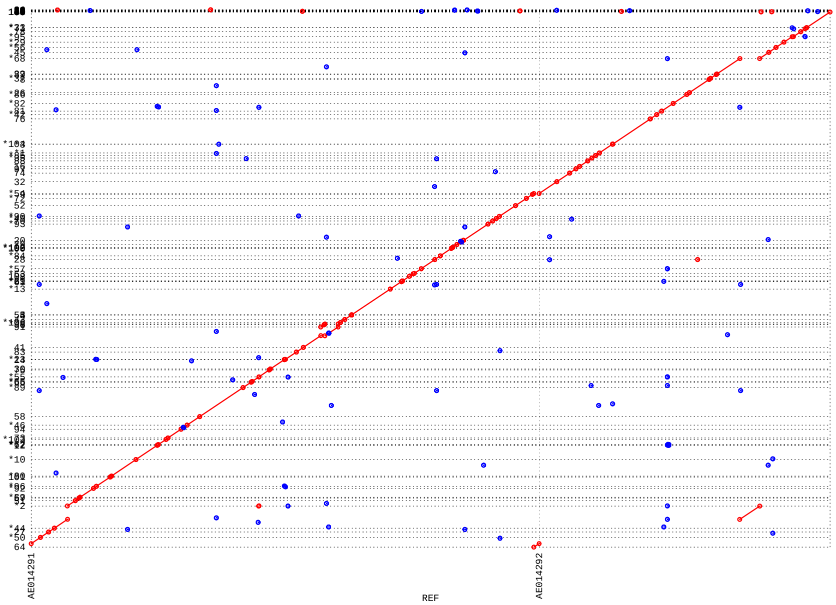

Figure 4: TIGR dotplot.

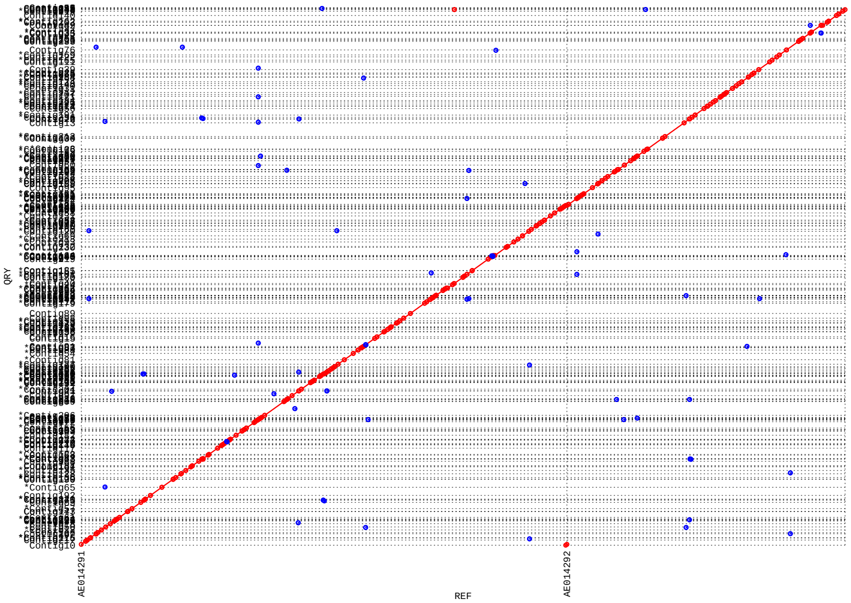

Figure 5: Euler dotplot.

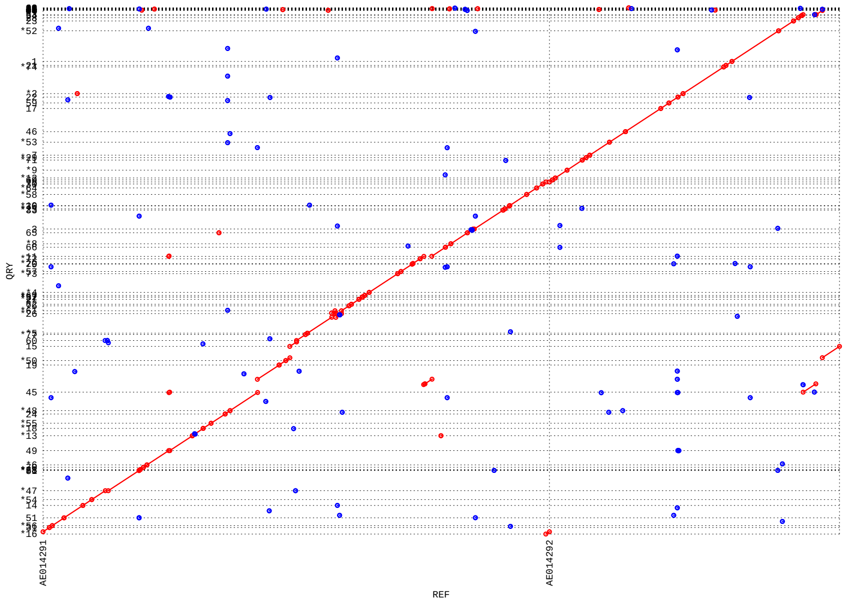

Figure 6: PCAP dotplot.

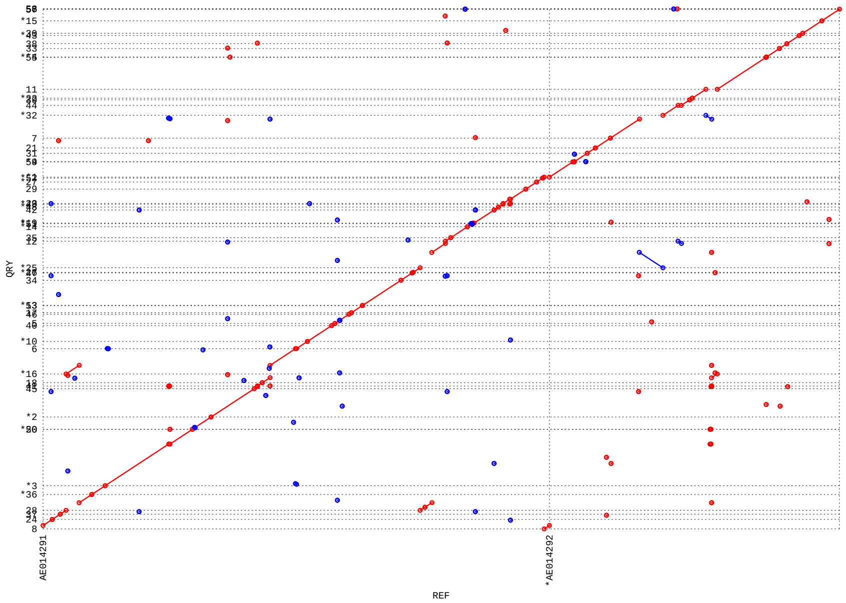

Figure 7: Sutta dotplot.

Staphylococcus epidermidis

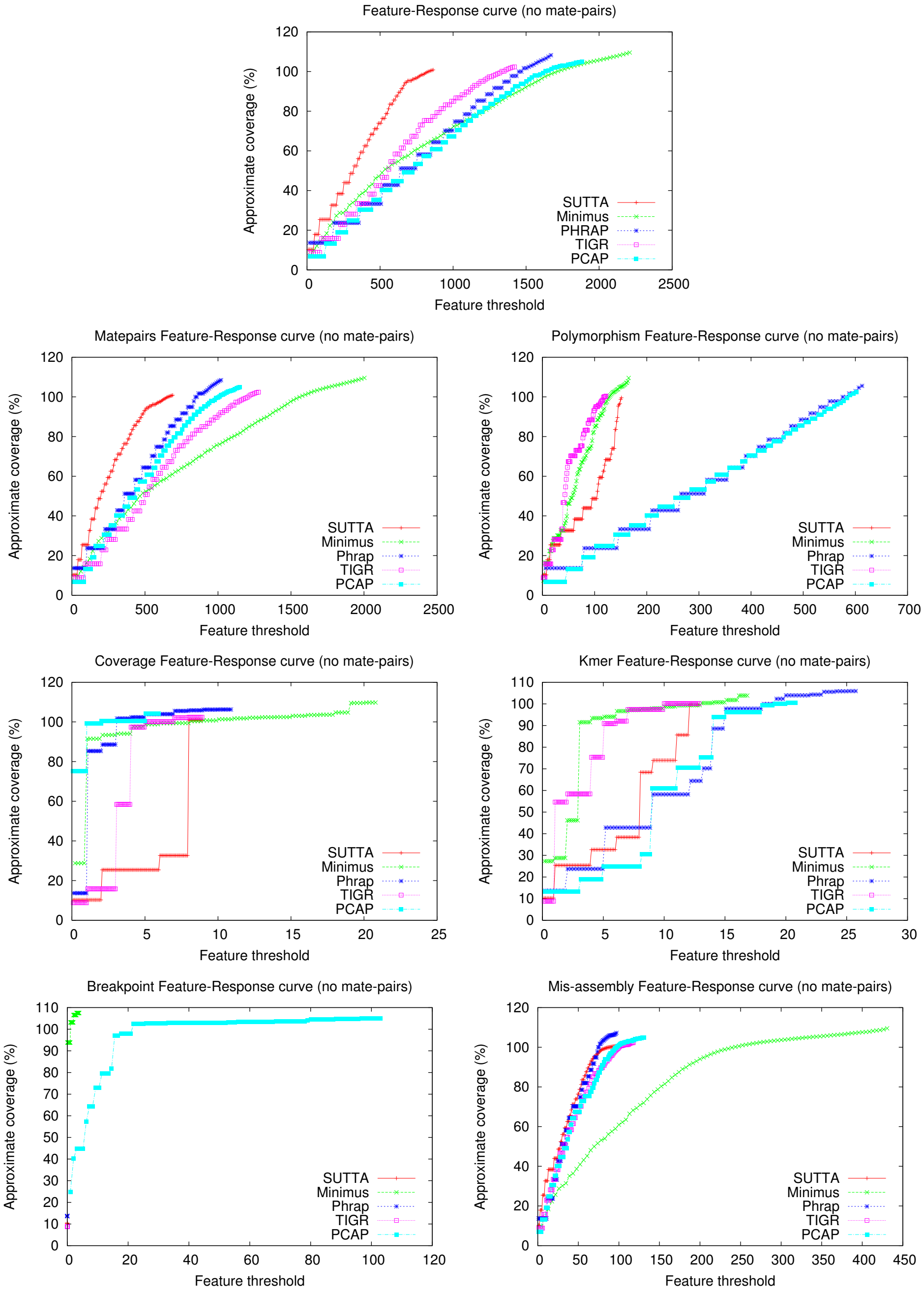

Figure 8: Feature-Response curves.

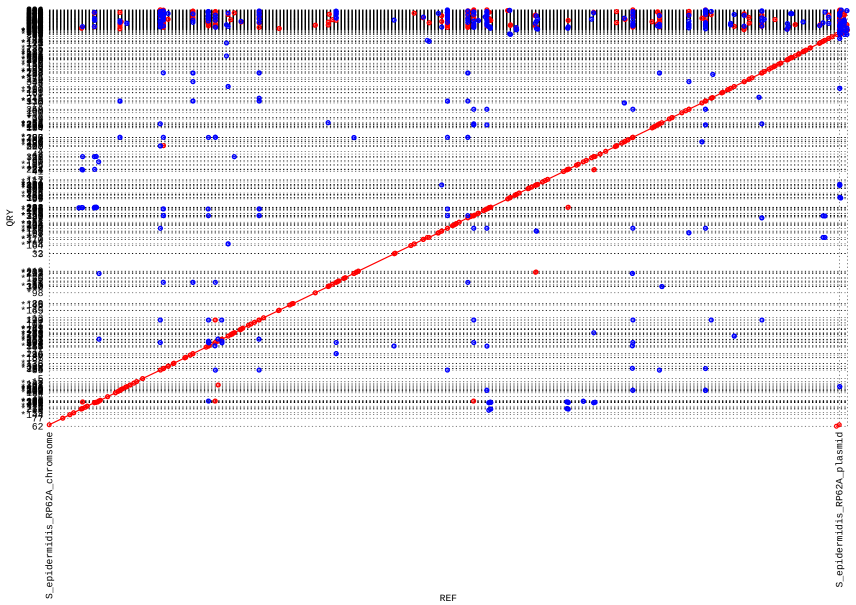

Figure 9: Minimus dotplot.

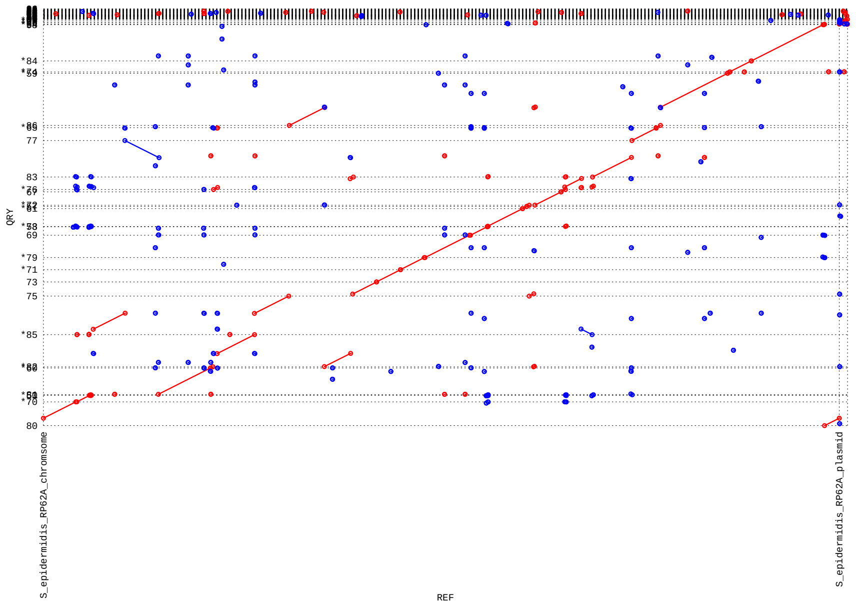

Figure 10: Phrap dotplot.

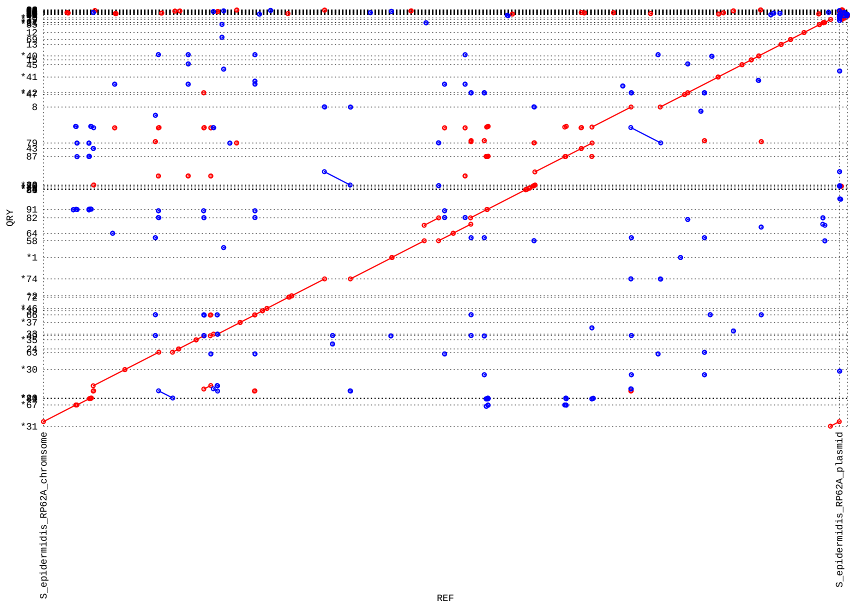

Figure 11: TIGR dotplot.

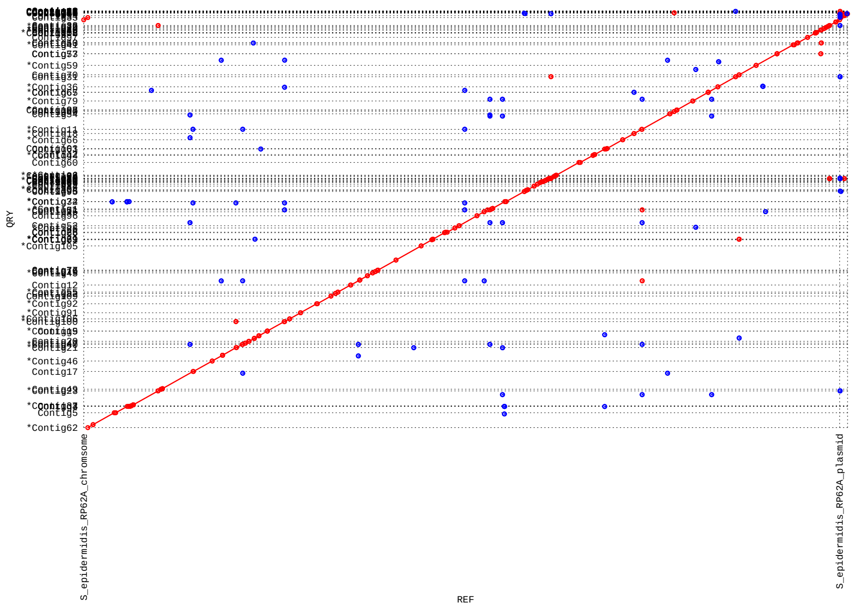

Figure 12: Euler dotplot.

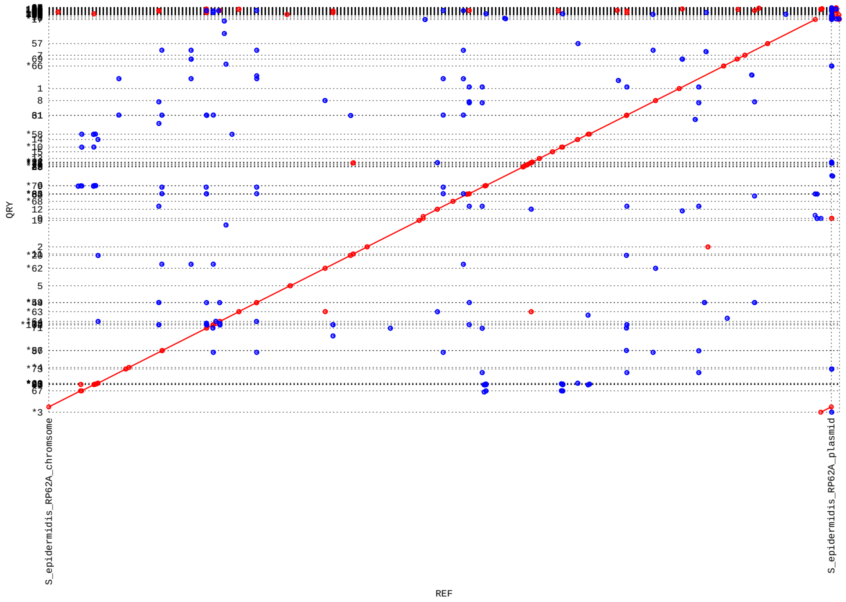

Figure 13: PCAP dotplot.

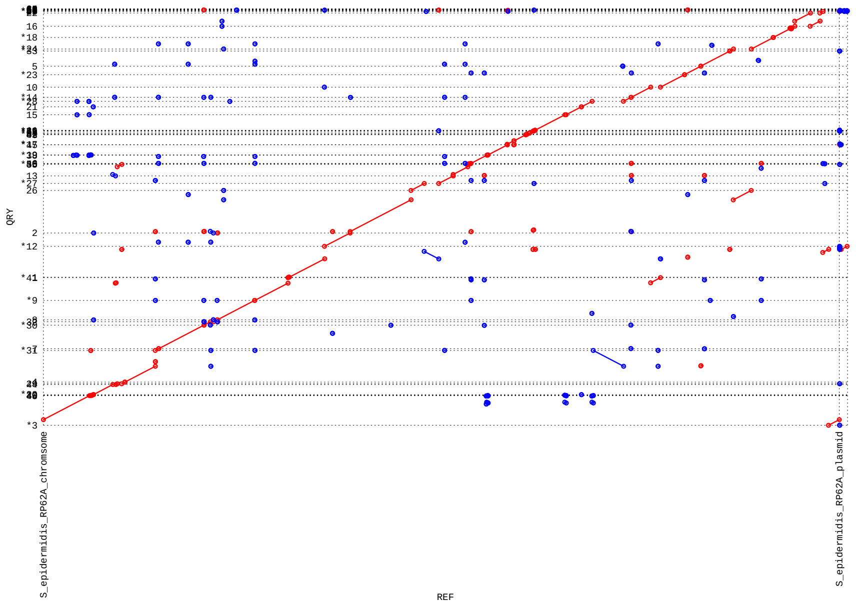

Figure 14: Sutta dotplot.

Wolbachia sp.

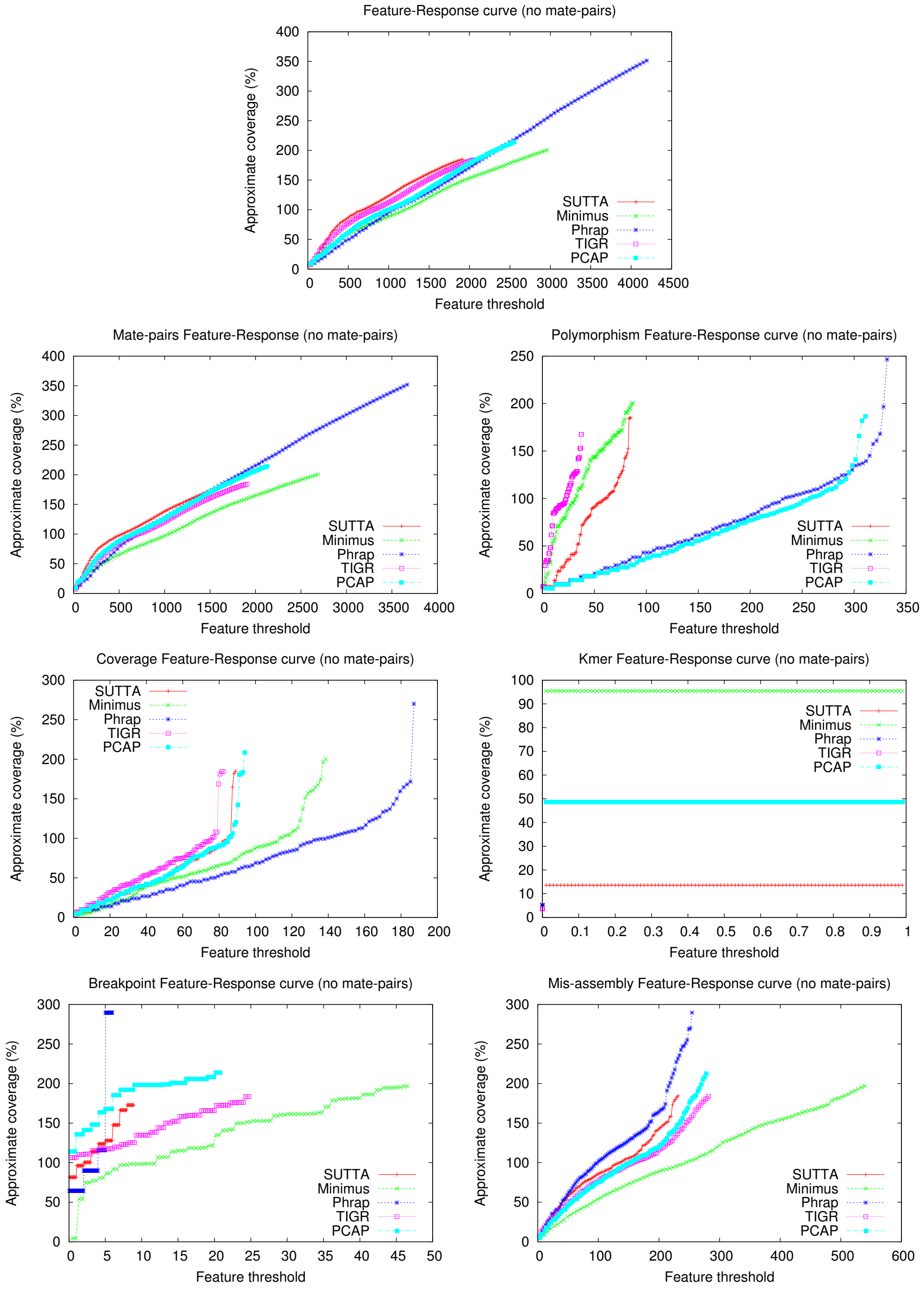

Figure 15: Feature-Response curves.

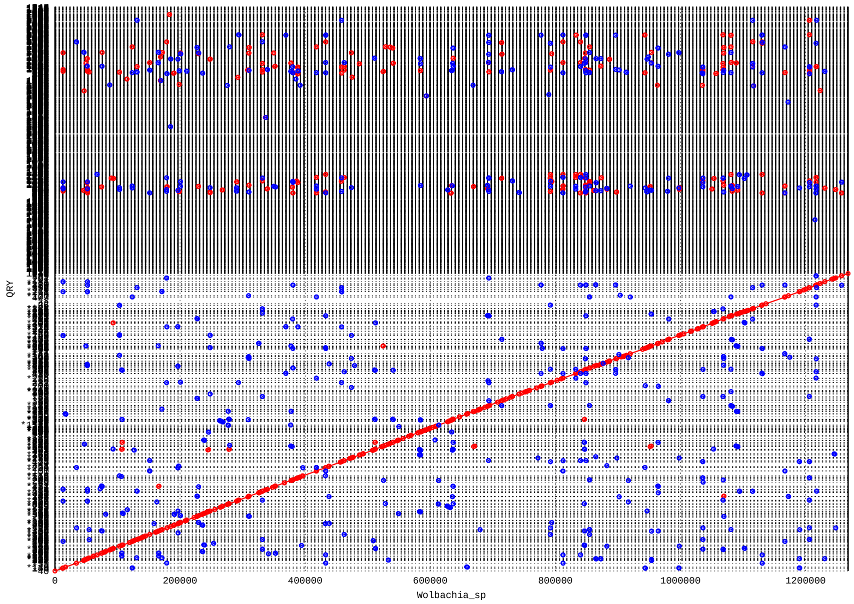

Figure 16: Minimus dotplot.

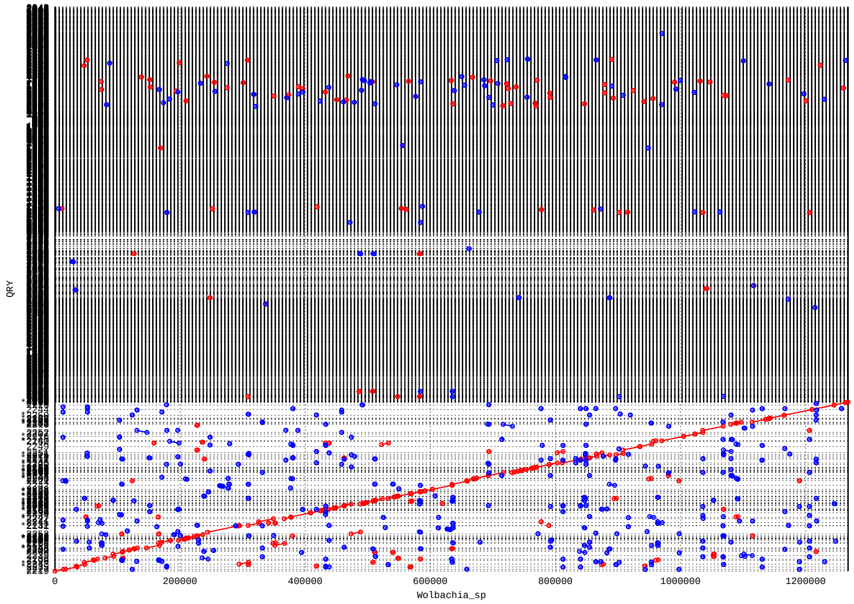

Figure 17: Phrap dotplot.

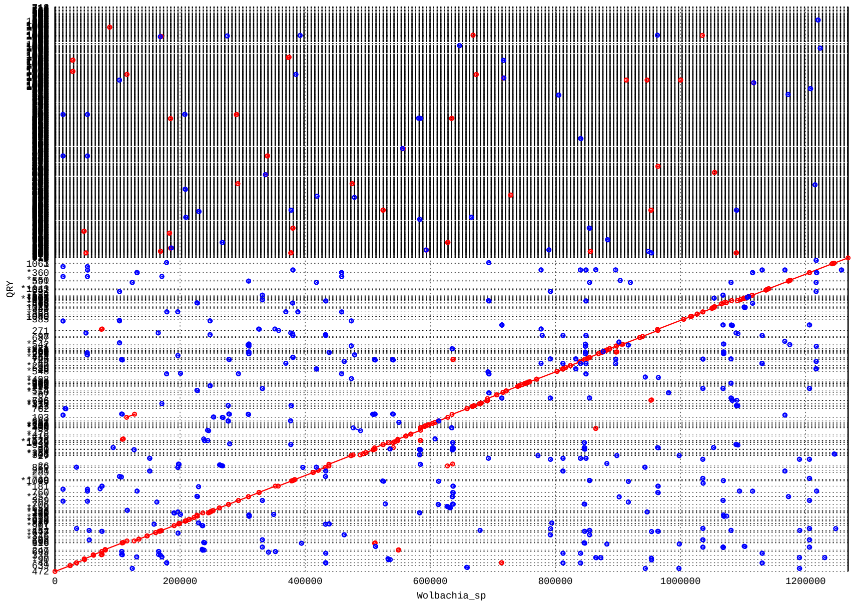

Figure 18: TIGR dotplot.

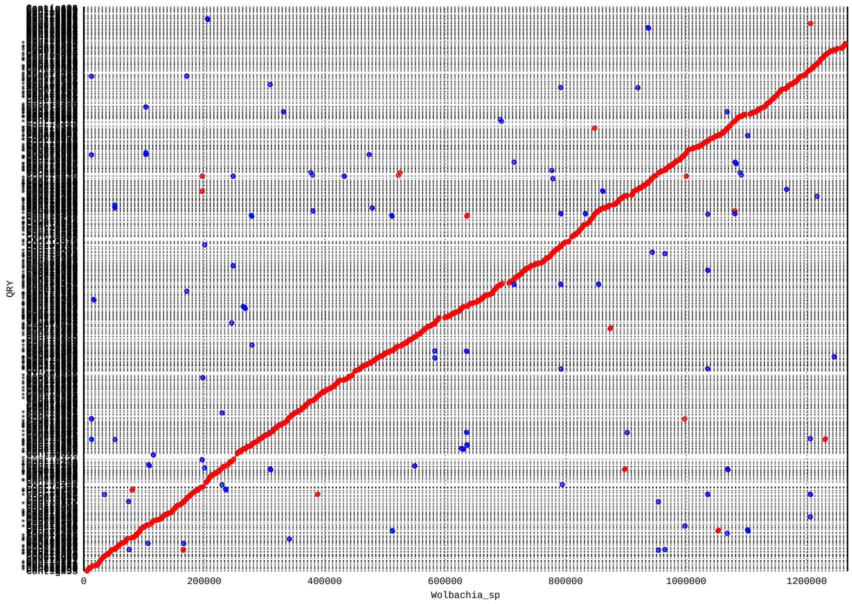

Figure 19: Euler dotplot.

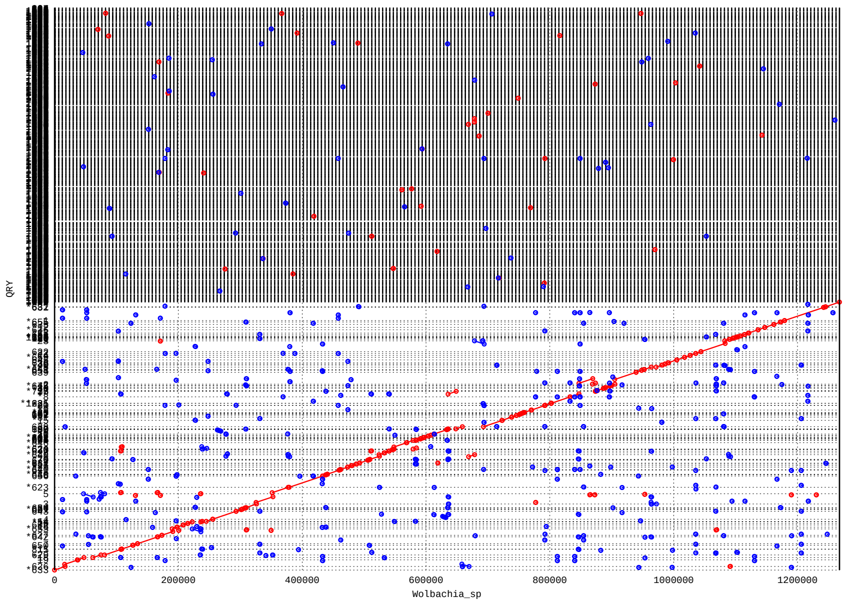

Figure 20: PCAP dotplot.

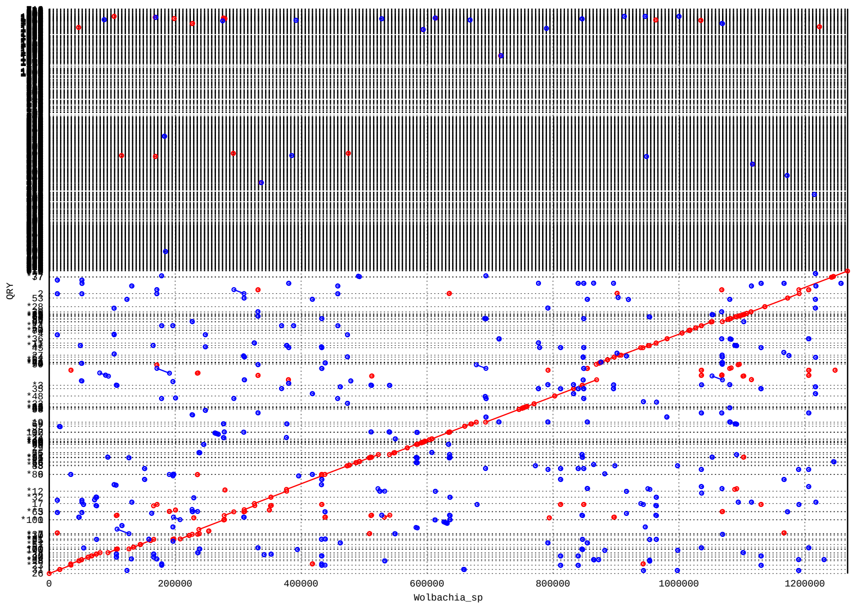

Figure 21: Sutta dotplot.

Chromosome Y 3Mbp section [35,000,001 - 38,000,000]

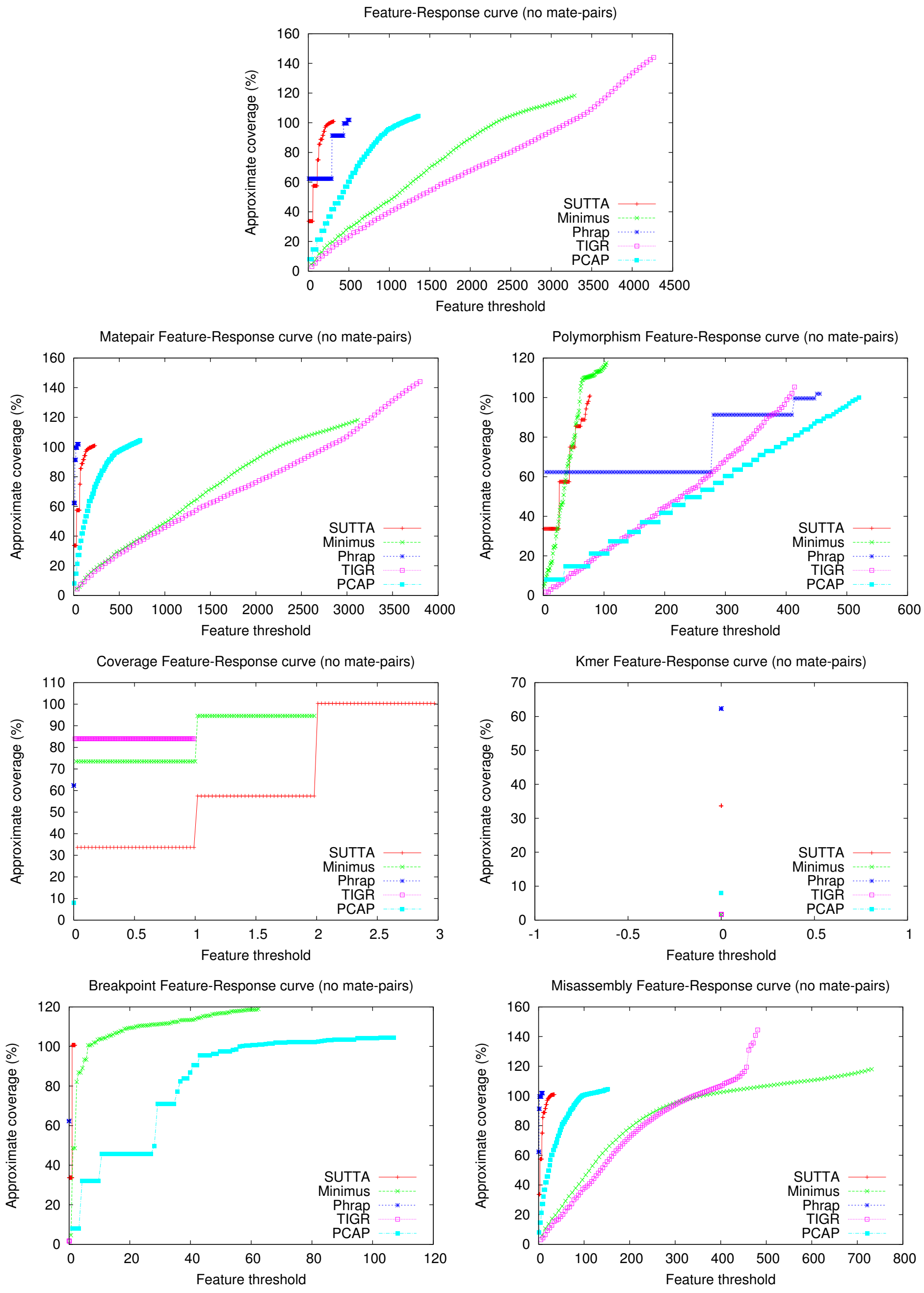

Figure 22: Feature-Response curves.

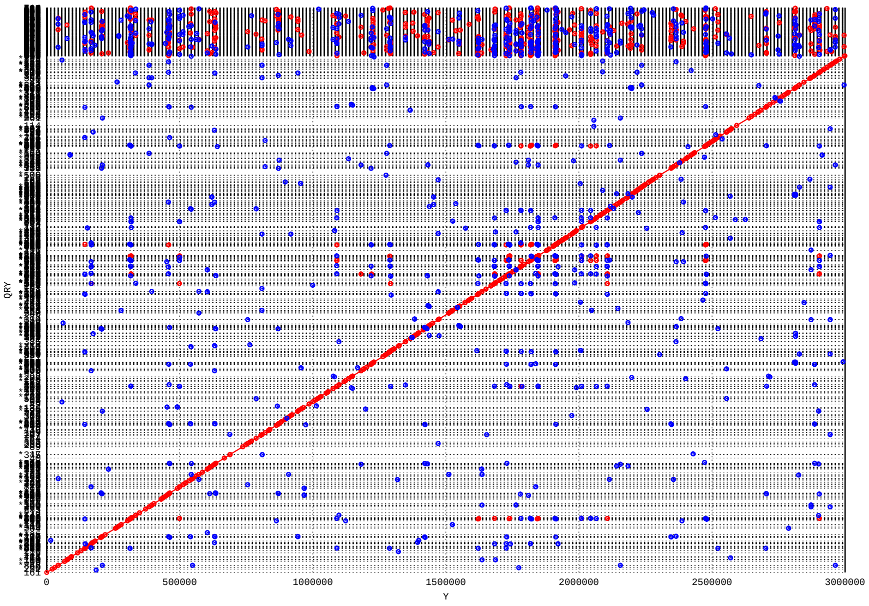

Figure 23: Minimus dotplot.

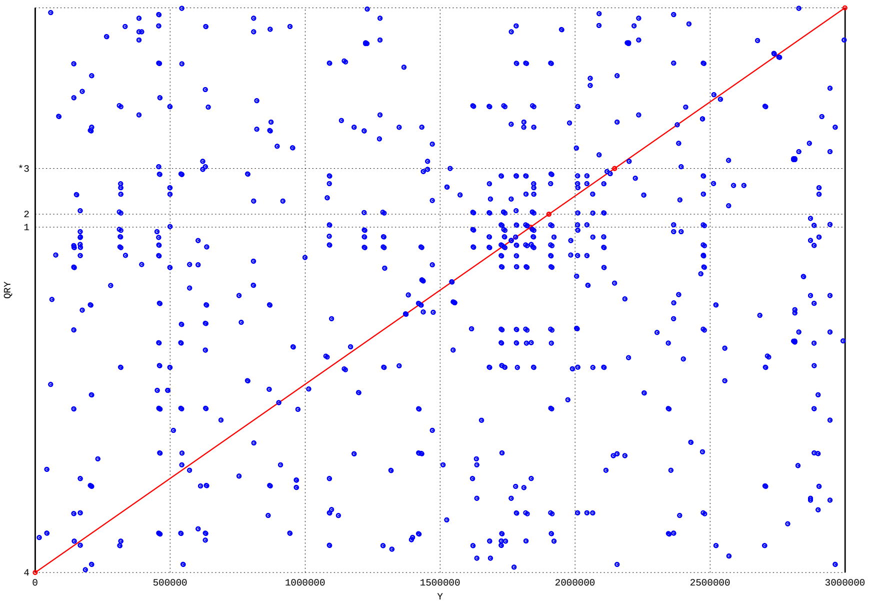

Figure 24: Phrap dotplot.

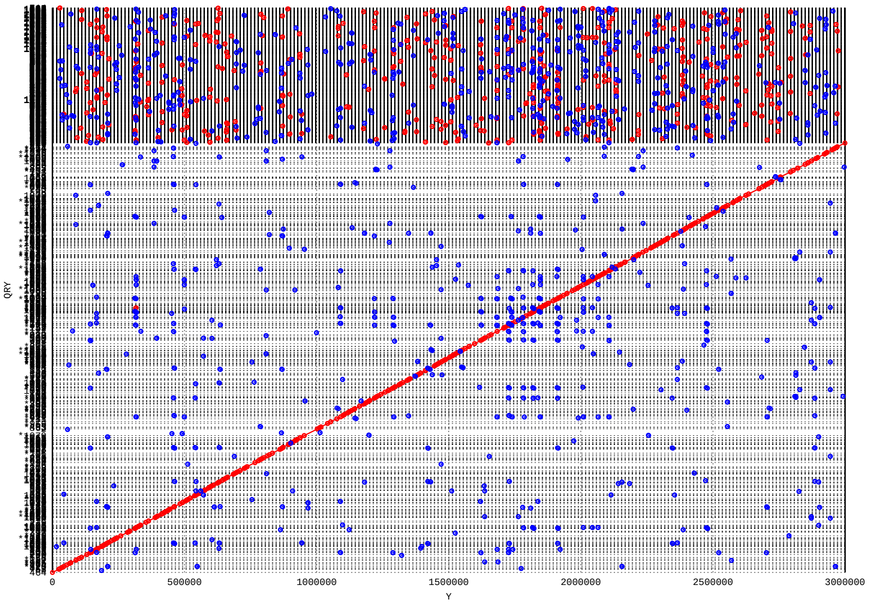

Figure 25: TIGR dotplot.

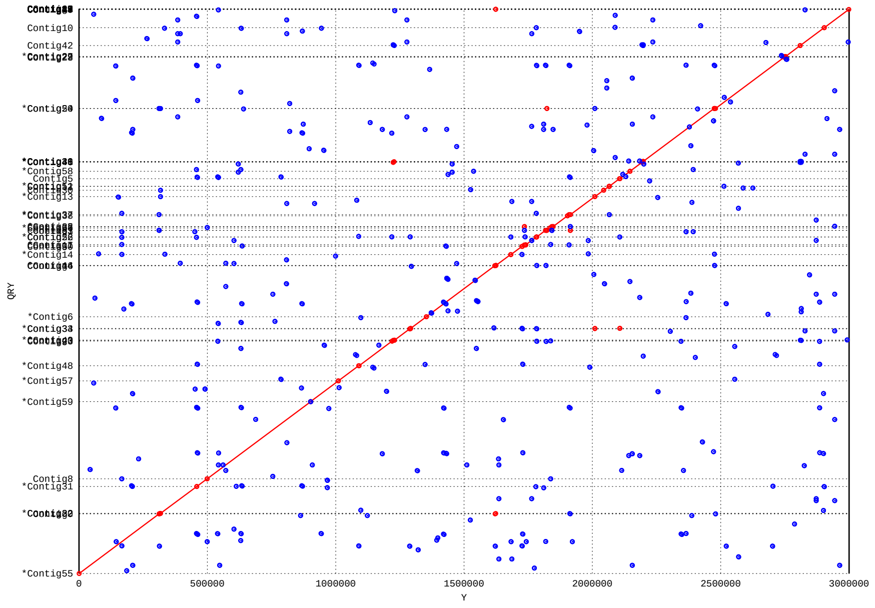

Figure 26: Euler dotplot.

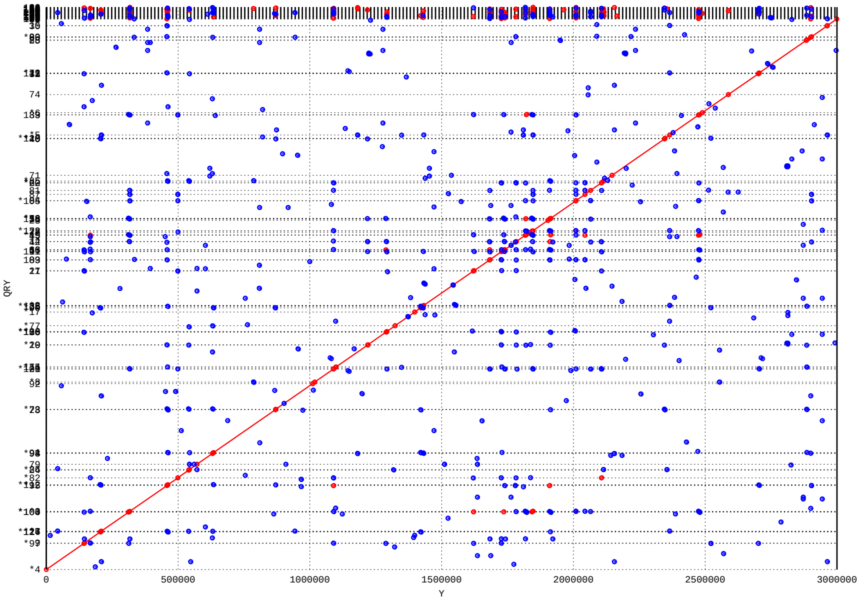

Figure 27: PCAP dotplot.

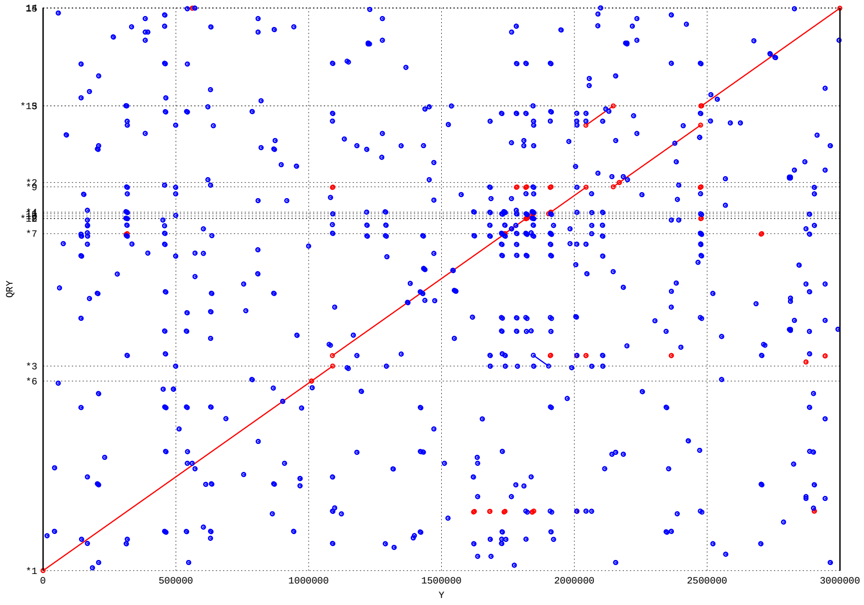

Figure 28: Sutta dotplot.

1.2 With Mate-Pairs constraints

Brucella suis

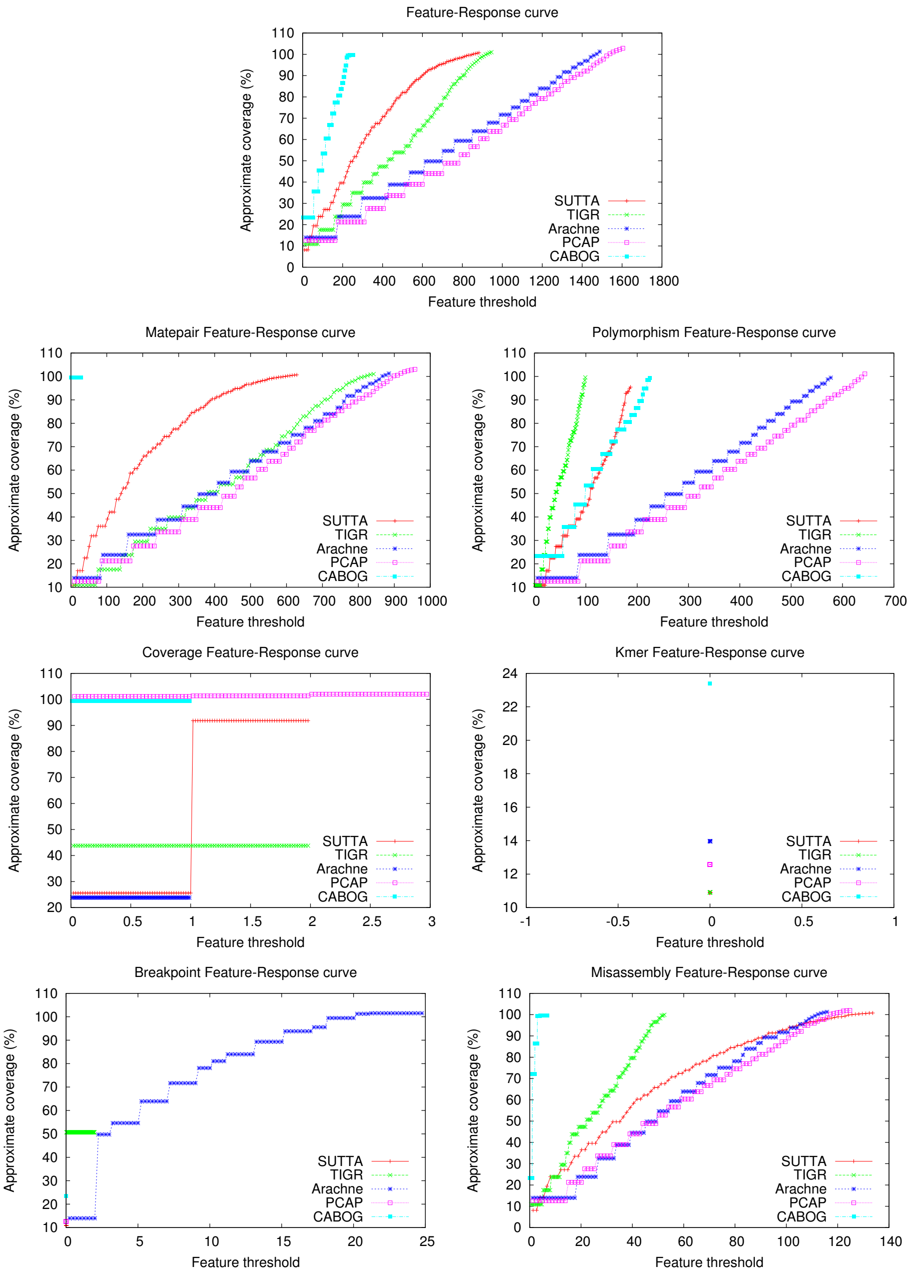

Figure 29: Feature-Response curves.

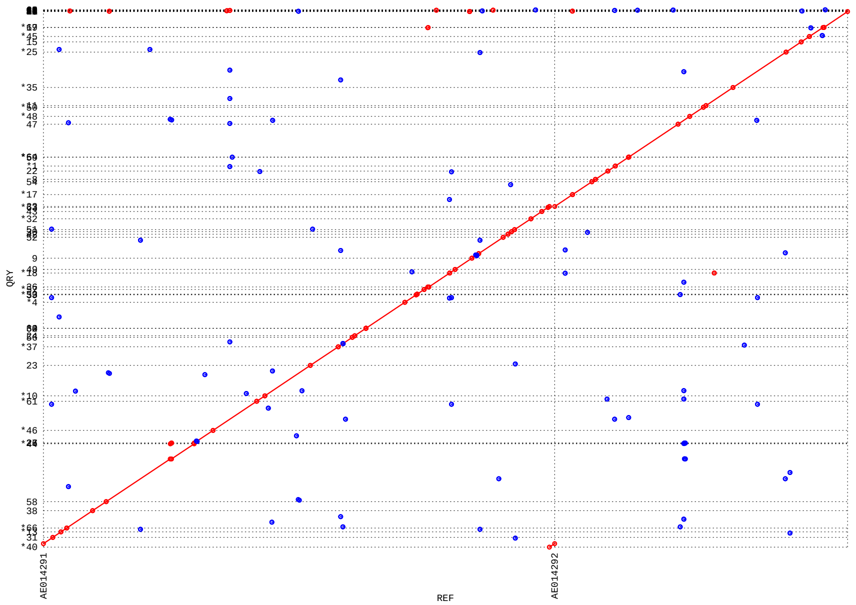

Figure 30: TIGR dotplot.

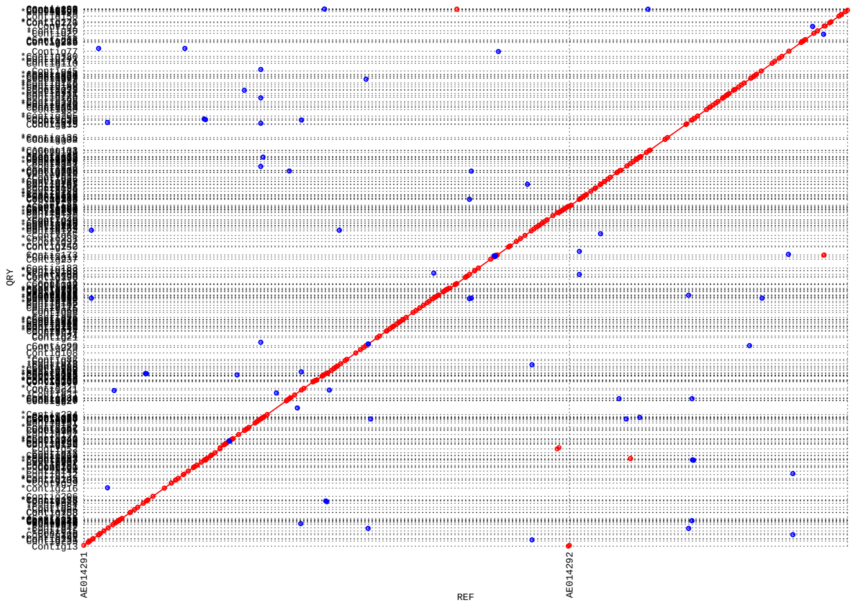

Figure 31: Euler dotplot.

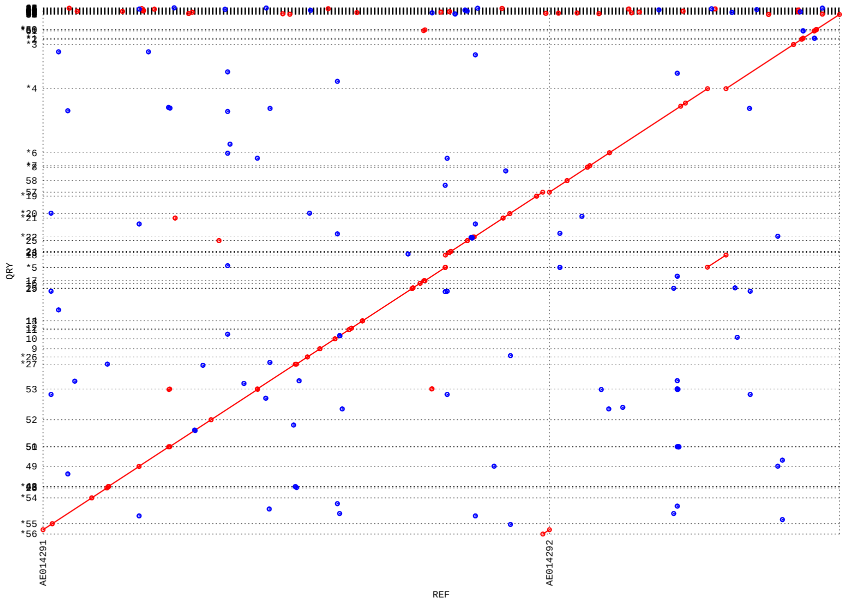

Figure 32: PCAP dotplot.

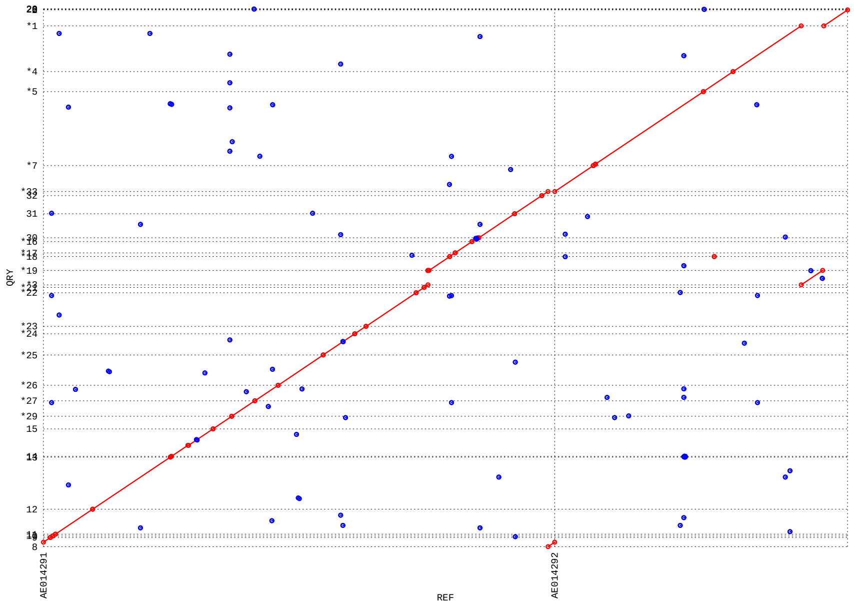

Figure 33: Arachne dotplot.

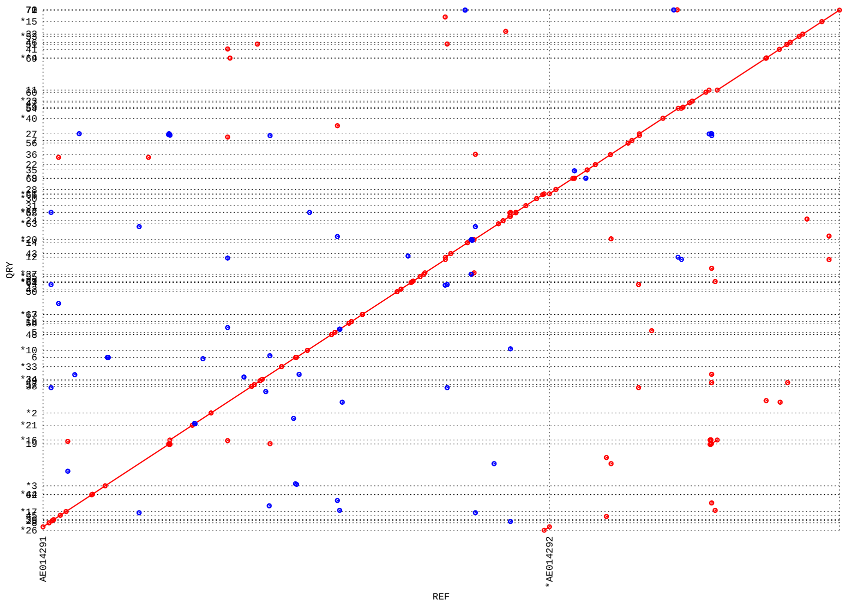

Figure 34: Sutta dotplot.

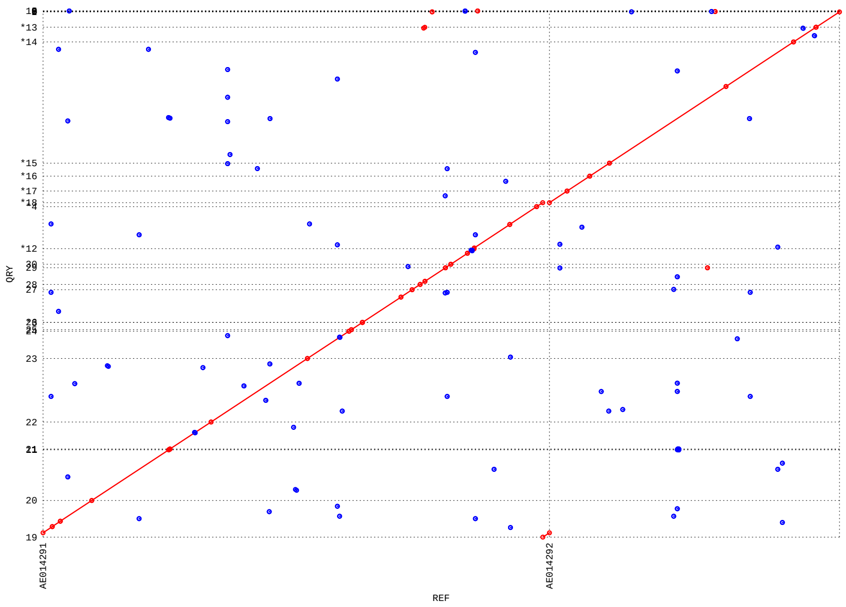

Figure 35: CABOG dotplot.

Staphylococcus epidermidis

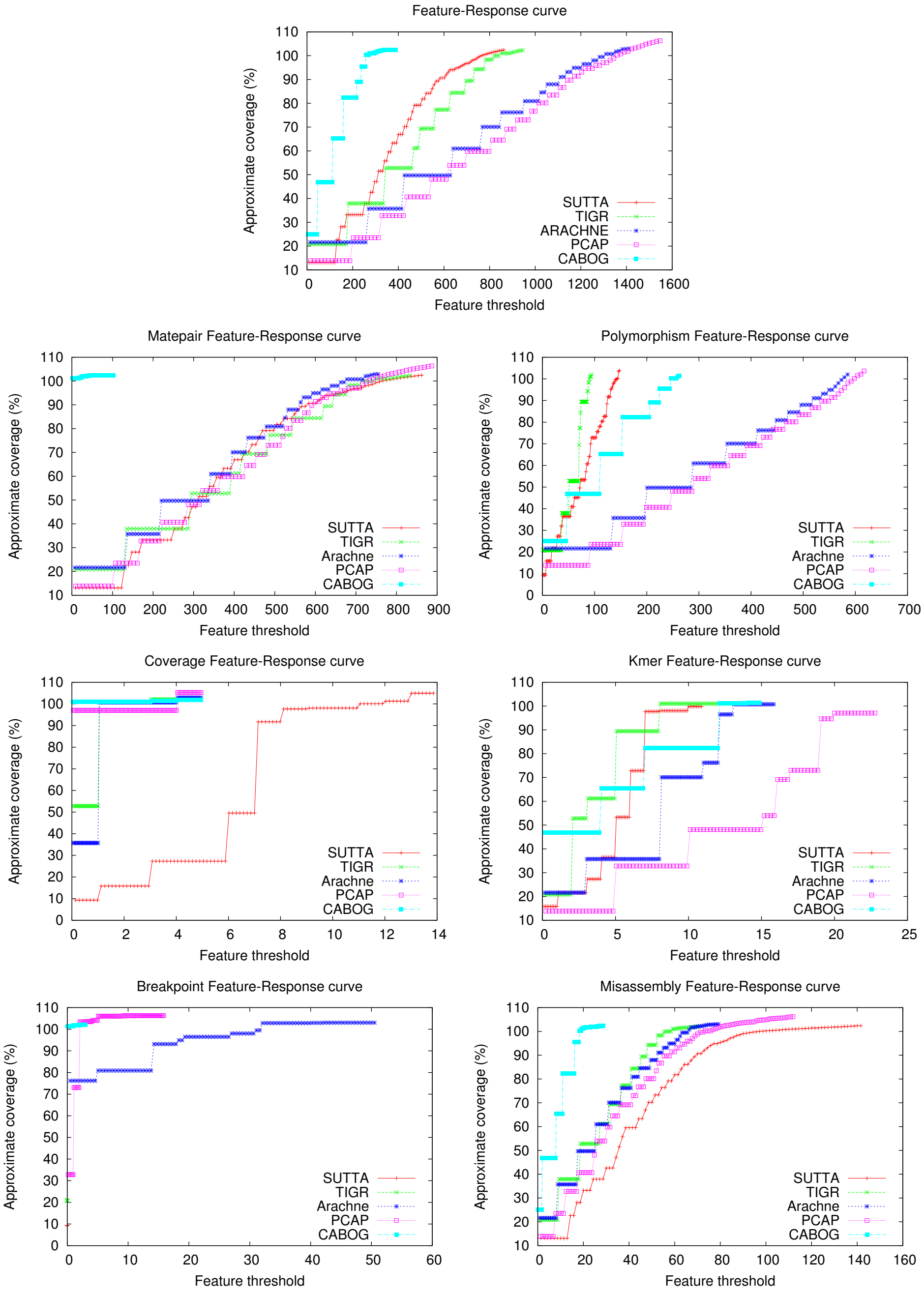

Figure 36: Feature-Response curves.

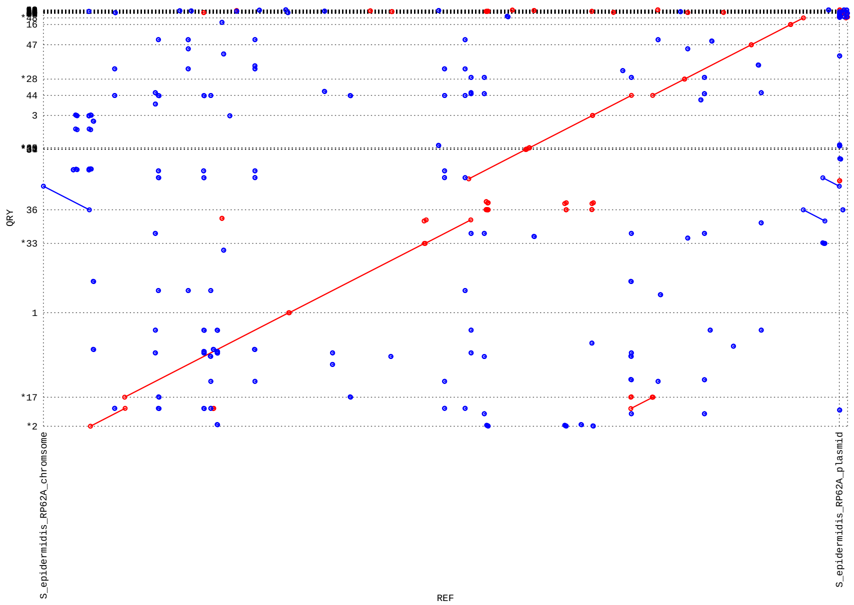

Figure 37: TIGR dotplot.

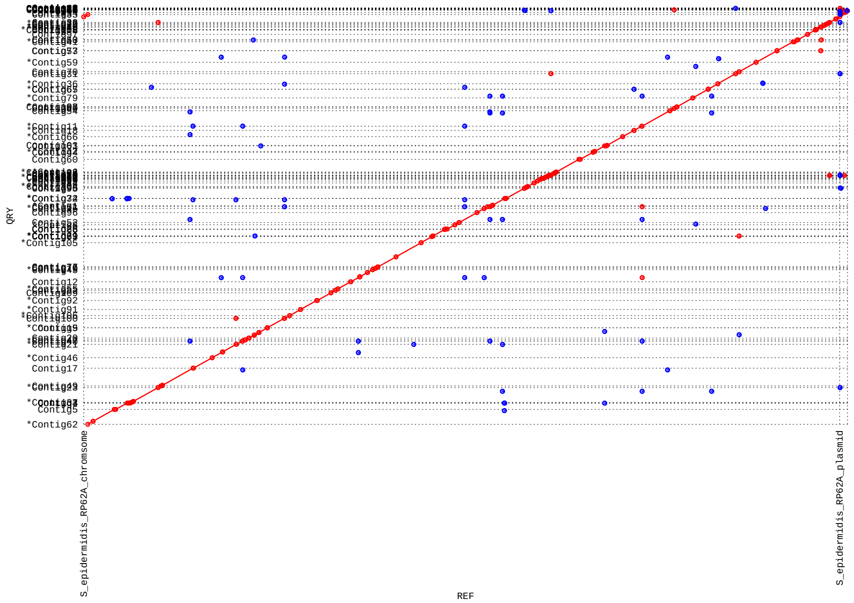

Figure 38: Euler dotplot.

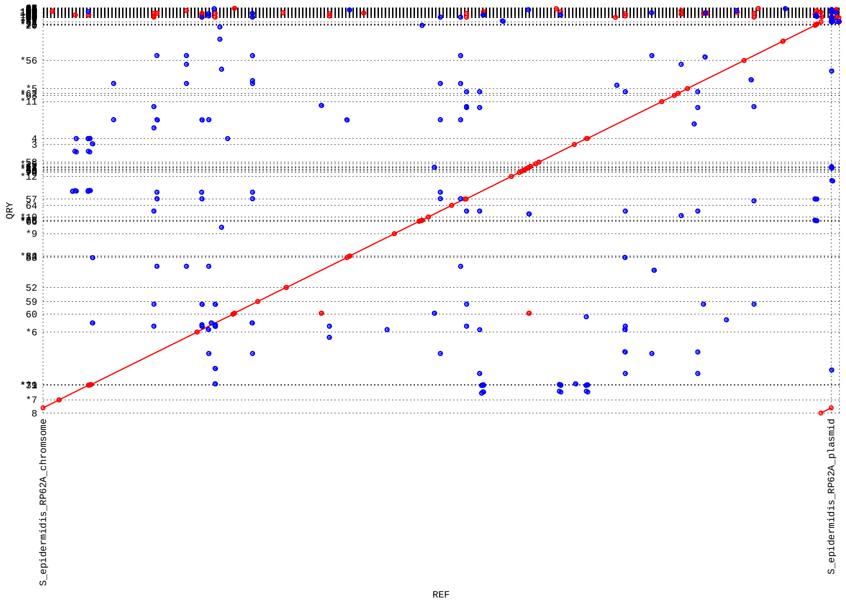

Figure 39: PCAP dotplot.

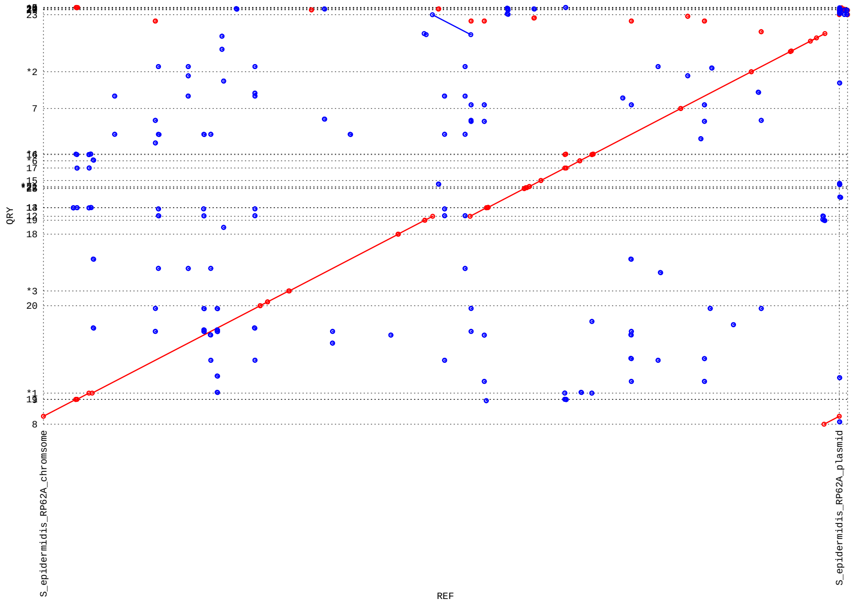

Figure 40: Arachne dotplot.

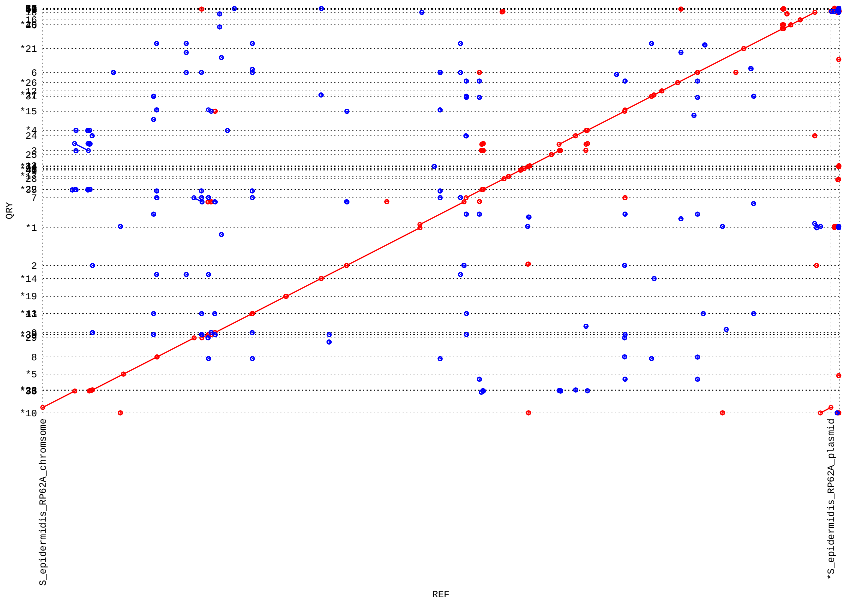

Figure 41: Sutta dotplot.

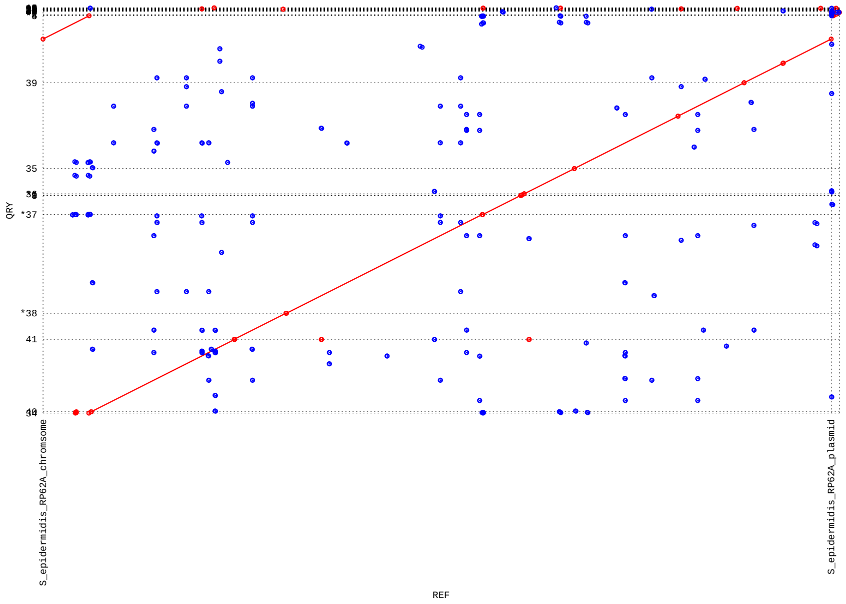

Figure 42: CABOG dotplot.

Wolbachia sp.

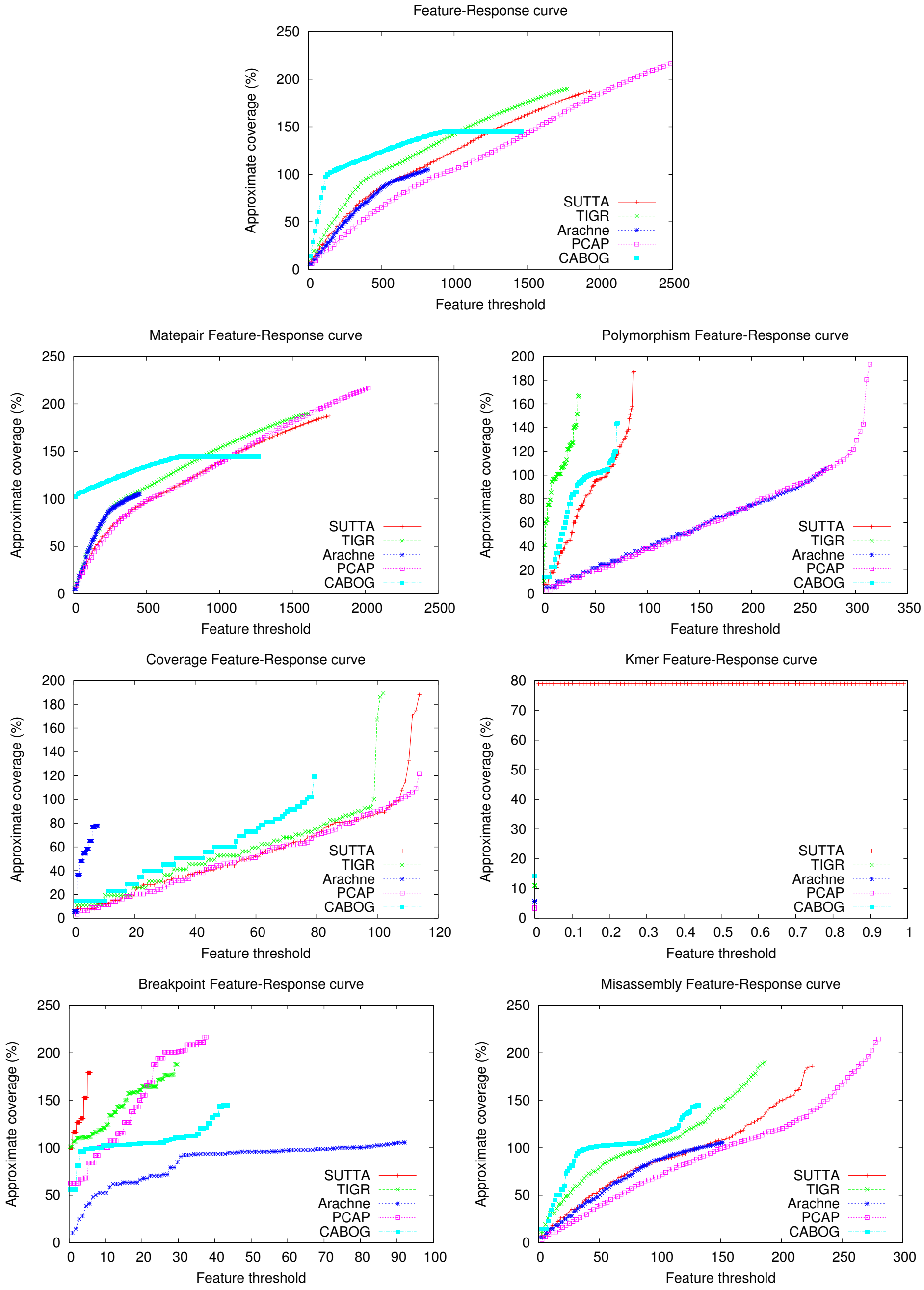

Figure 43: Feature-Response curve.

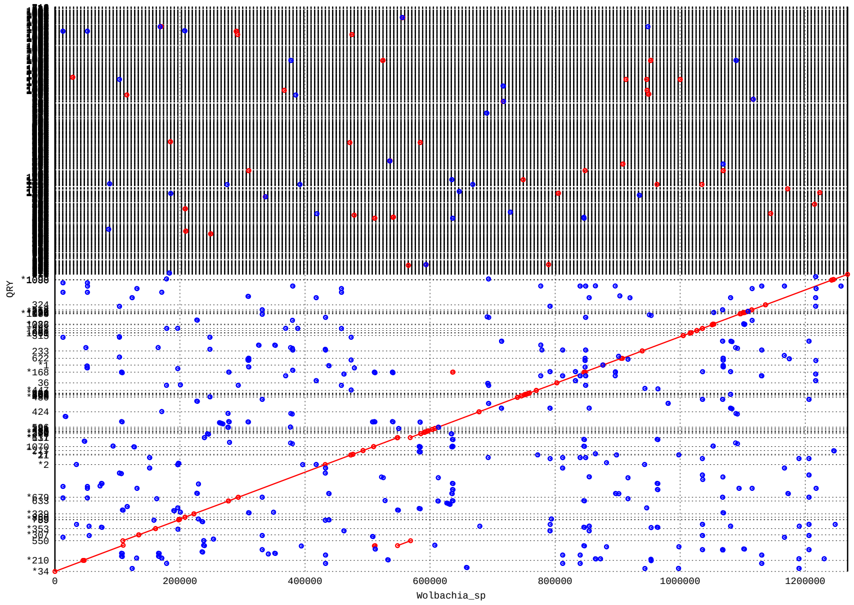

Figure 44: TIGR dotplot.

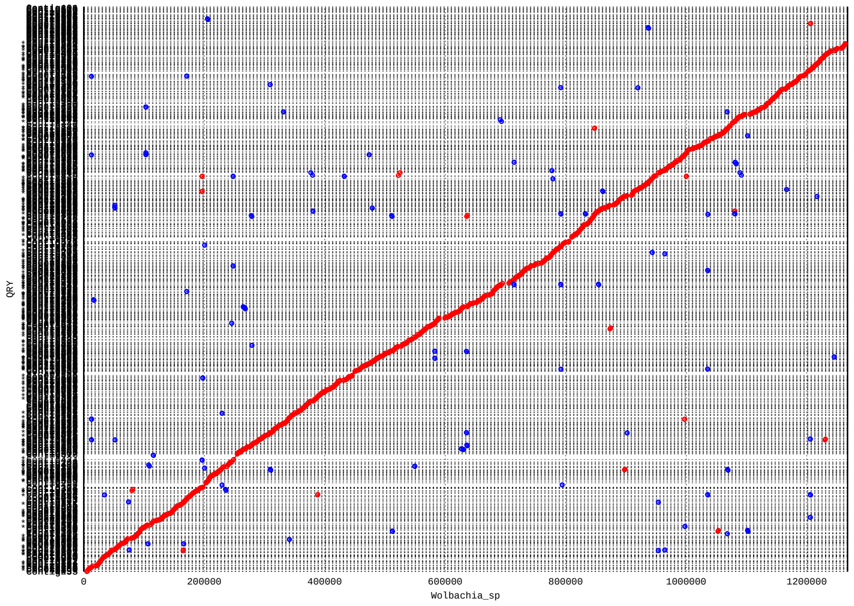

Figure 45: Euler dotplot.

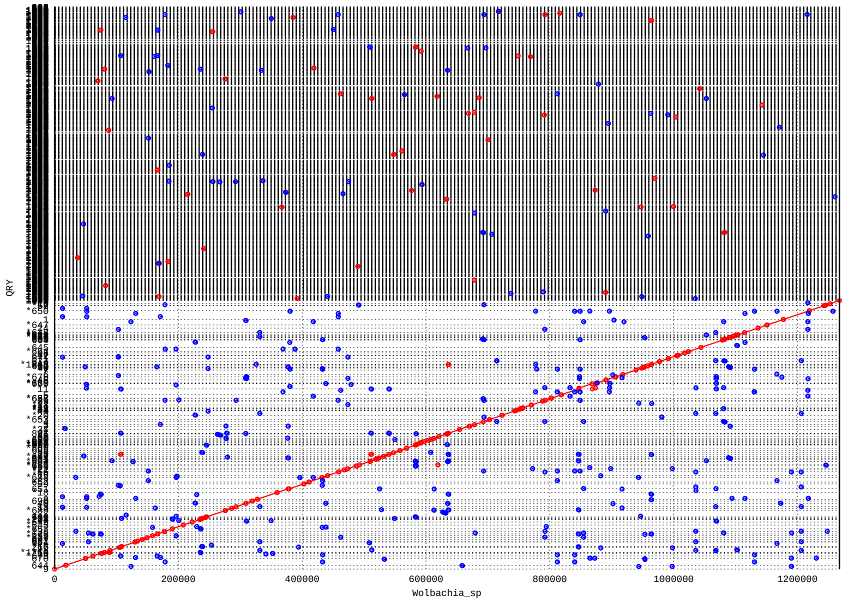

Figure 46: PCAP dotplot.

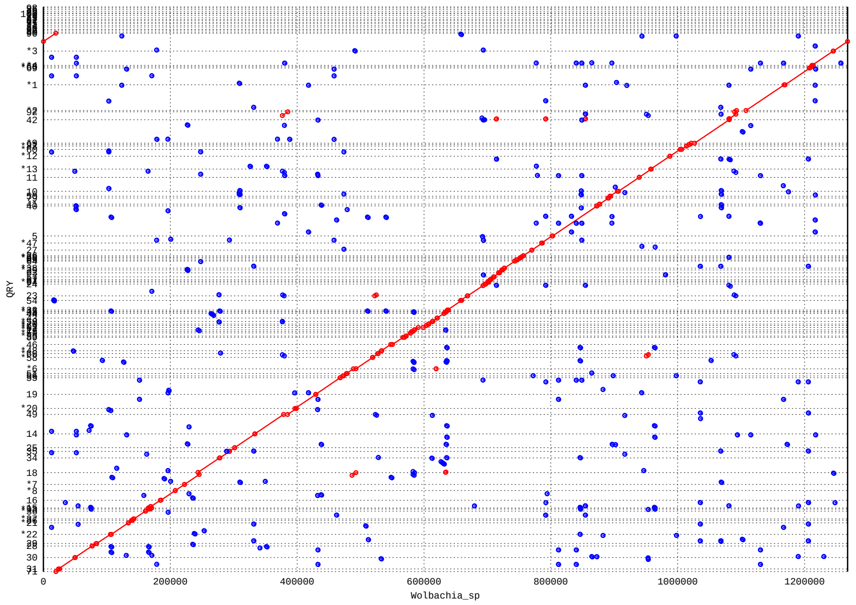

Figure 47: Arachne dotplot.

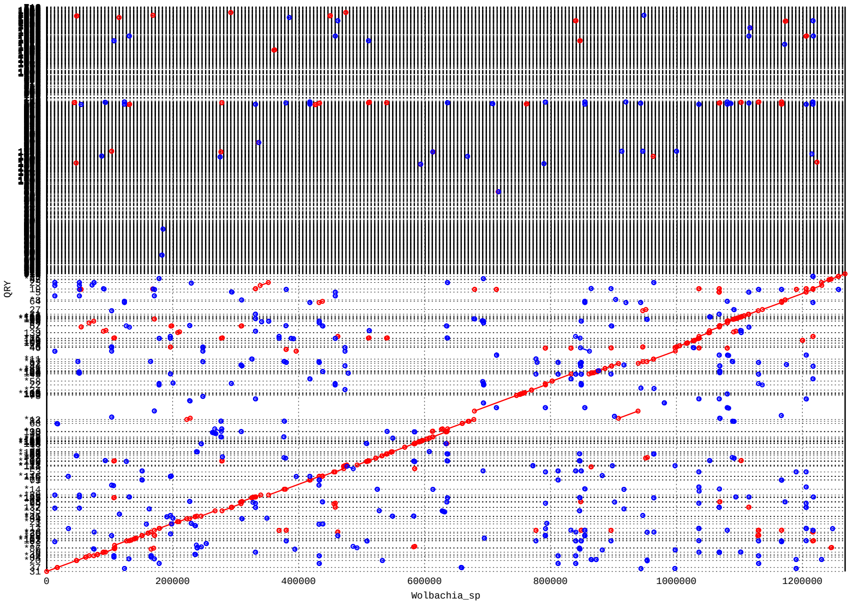

Figure 48: Sutta dotplot.

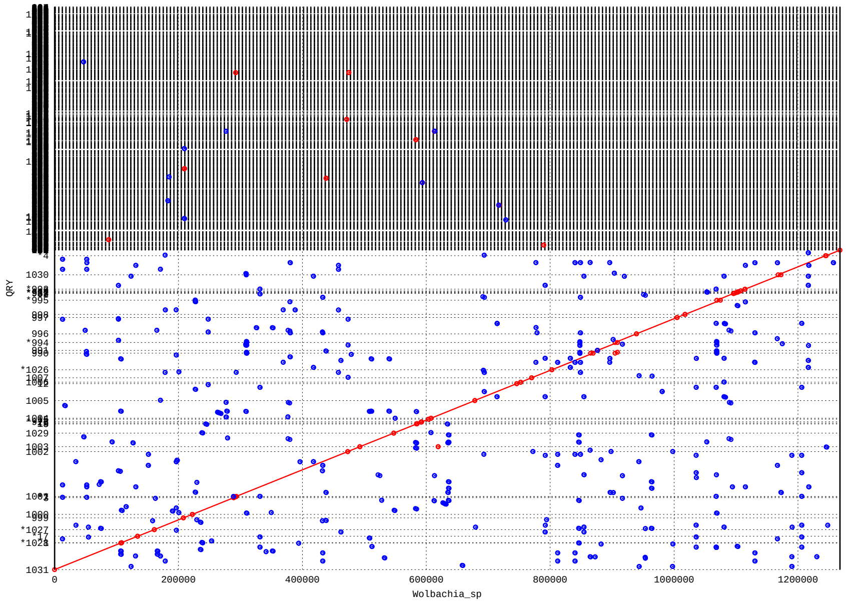

Figure 49: CABOG dotplot.

Chromosome Y 3Mbp section [35,000,001 - 38,000,000]

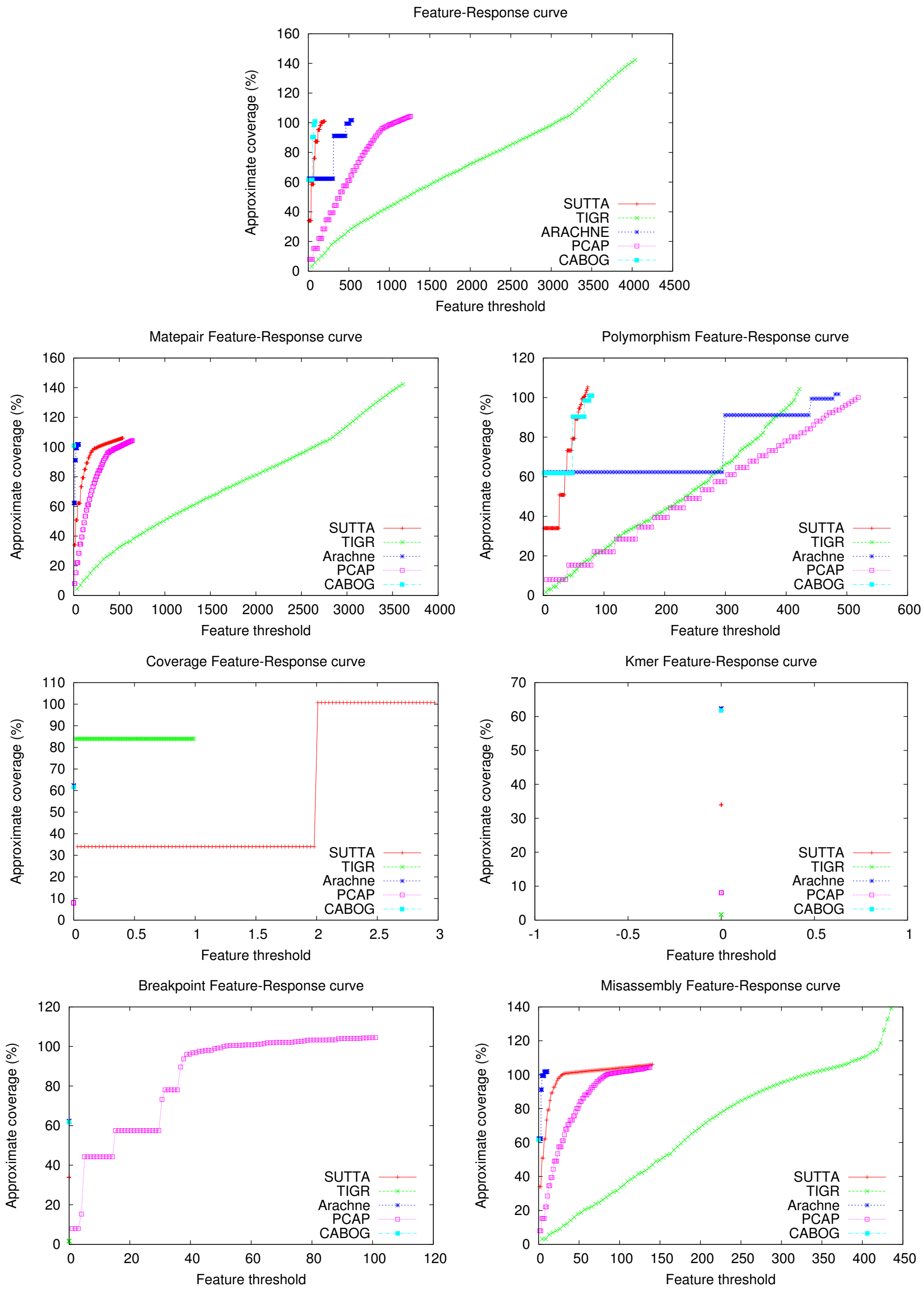

Figure 50: Feature-Response curves.

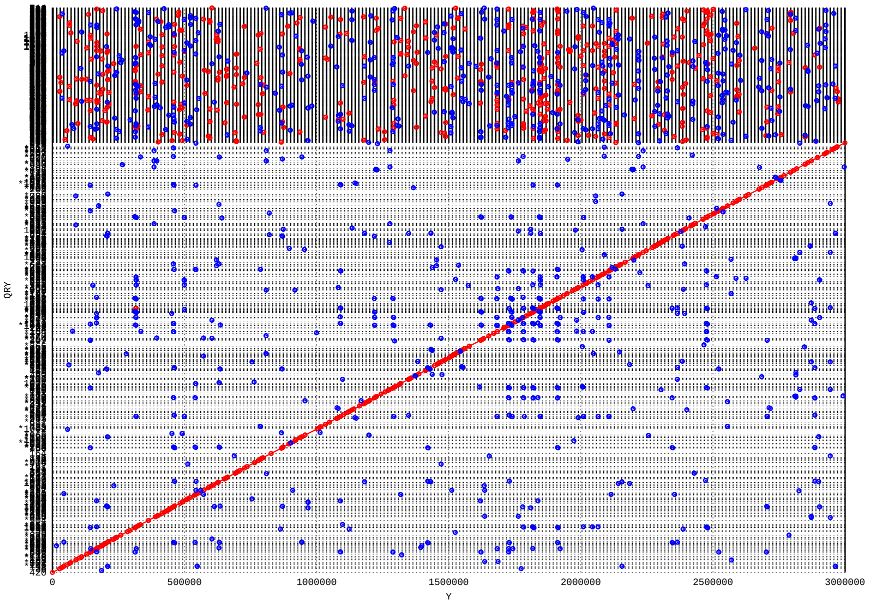

Figure 51: TIGR dotplot.

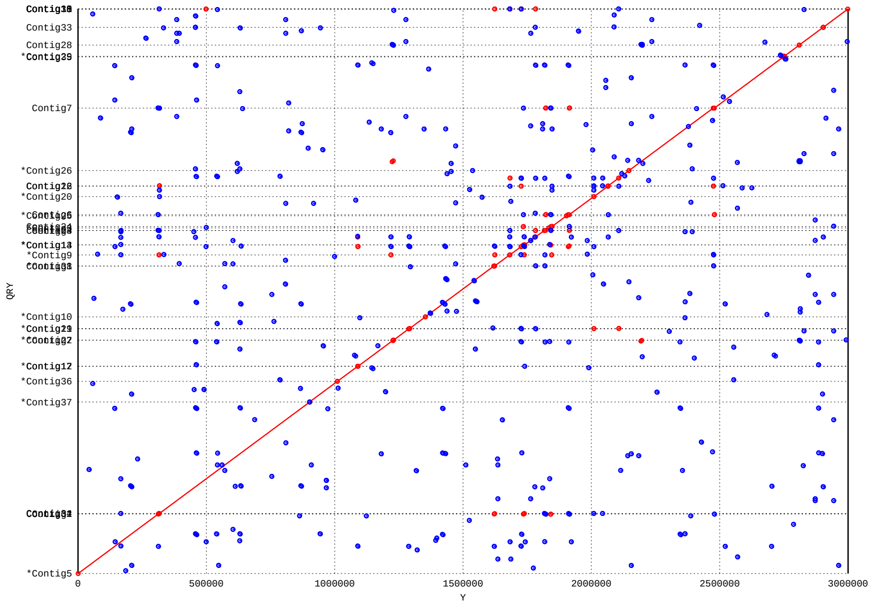

Figure 52: Euler dotplot.

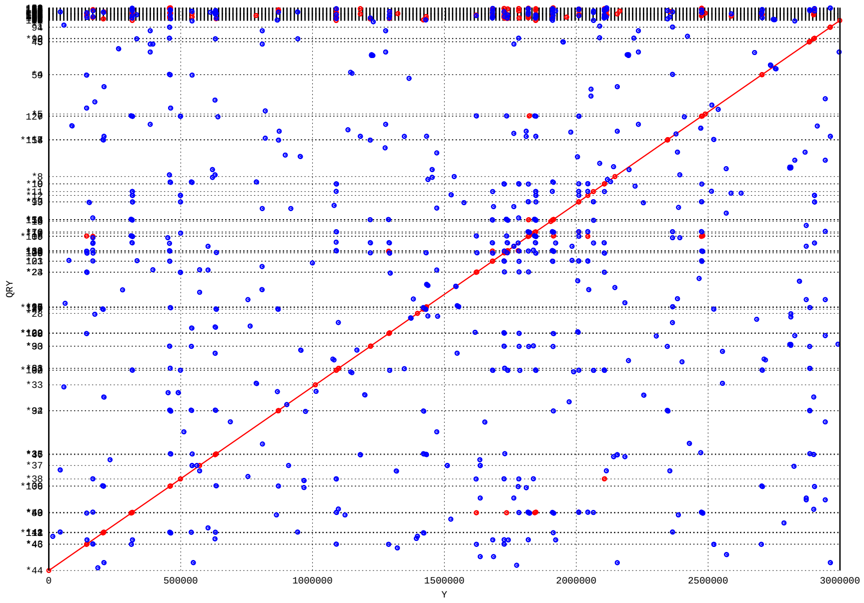

Figure 53: PCAP dotplot.

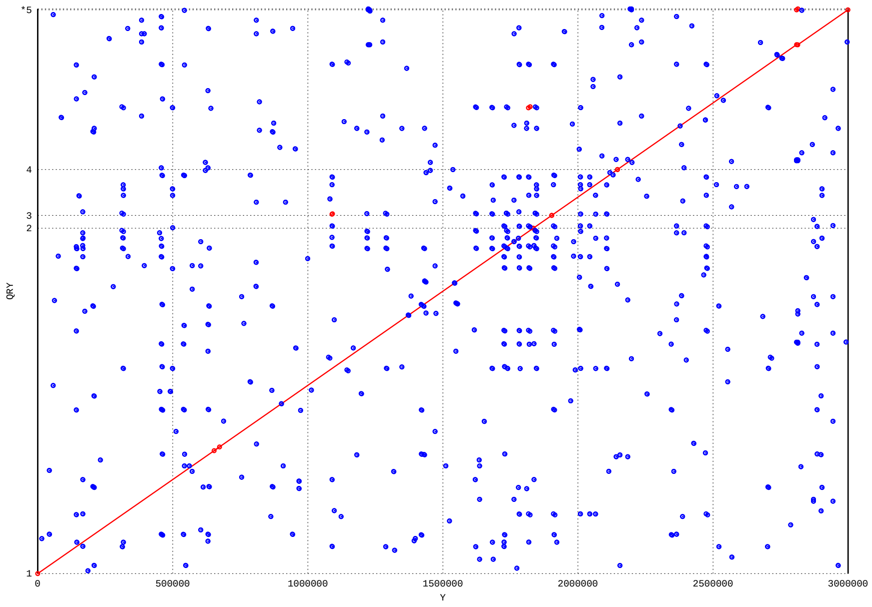

Figure 54: Arachne dotplot.

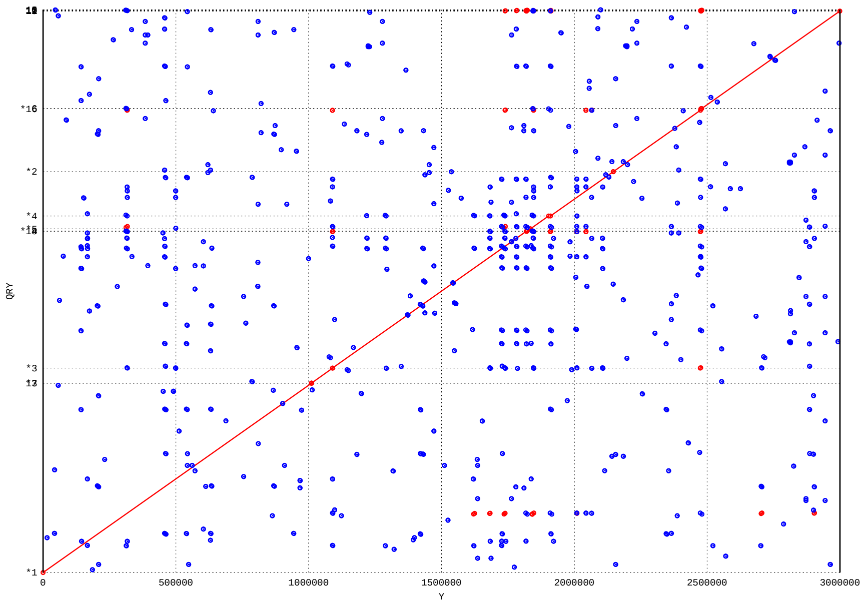

Figure 55: Sutta dotplot.

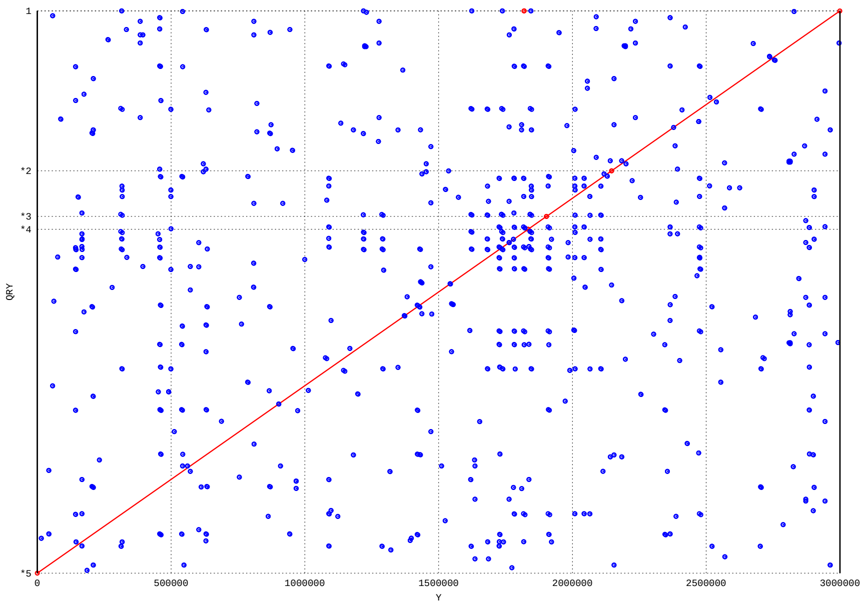

Figure 56: CABOG dotplot.

2 Short Reads

2.1 With Mate-Pairs constraints

Escherichia coli

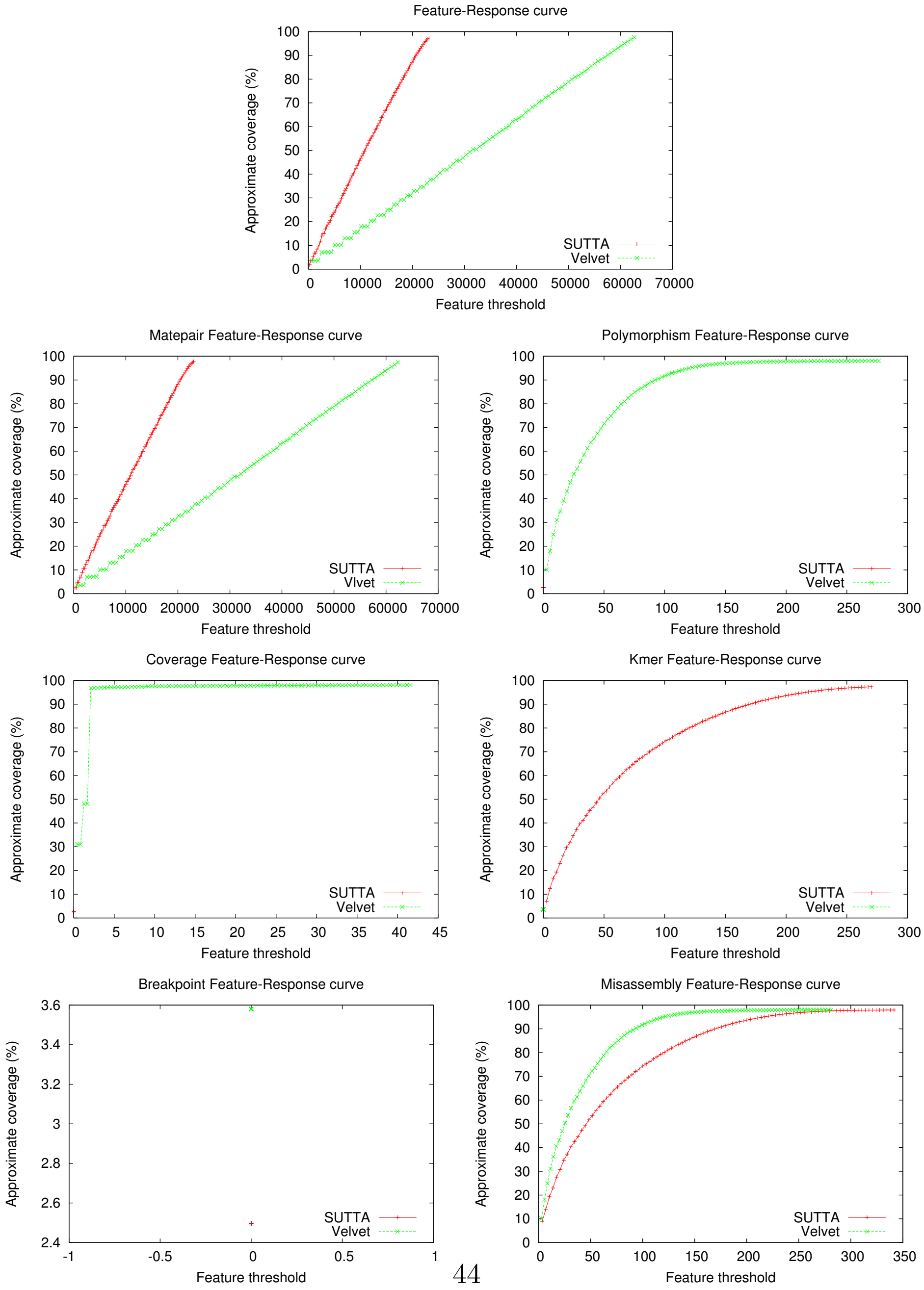

Figure 57: Feature-Response curves.

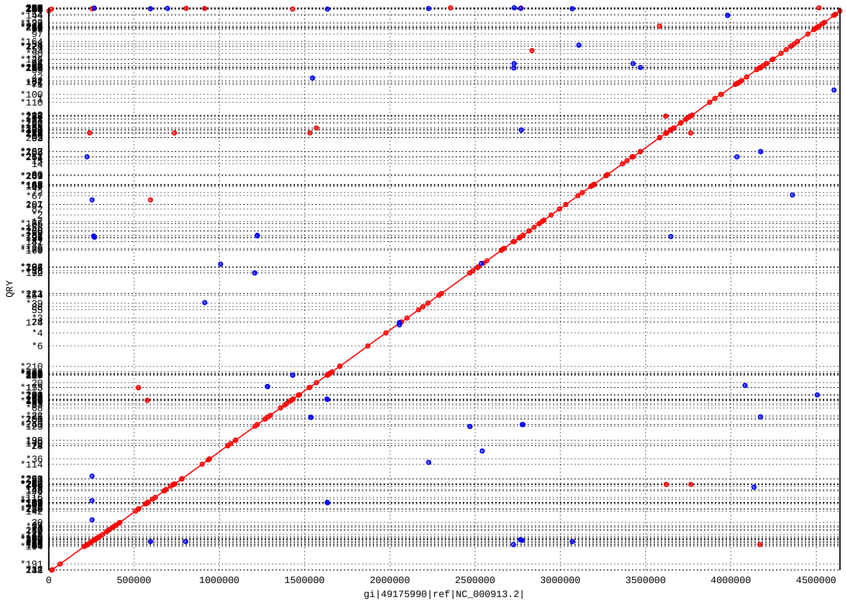

Figure 58: Velvet dotplot.

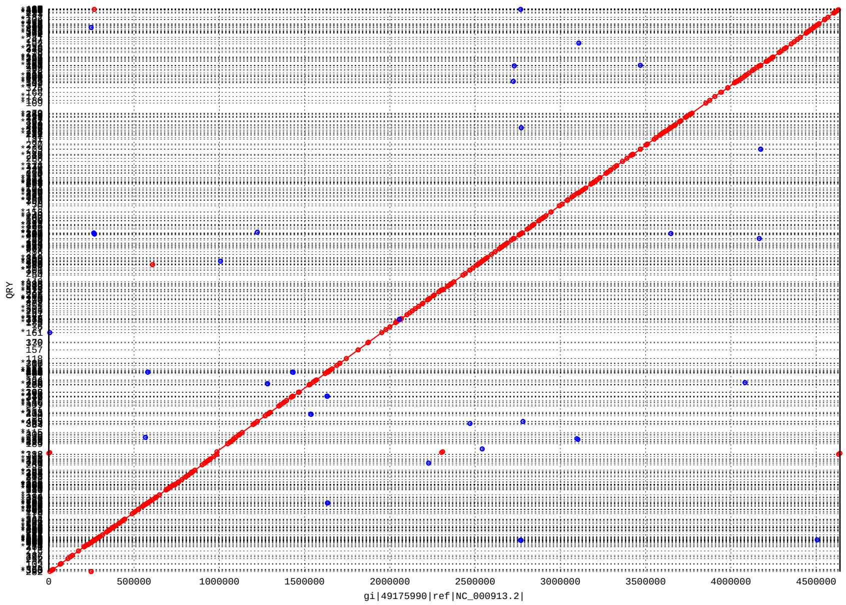

Figure 59: SUTTA dotplot.

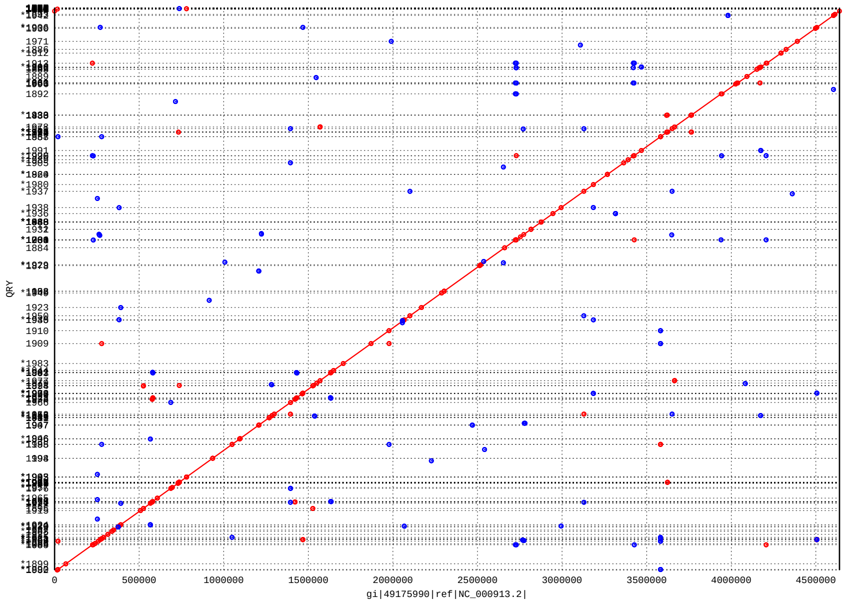

Figure 60: ABySS dotplot.

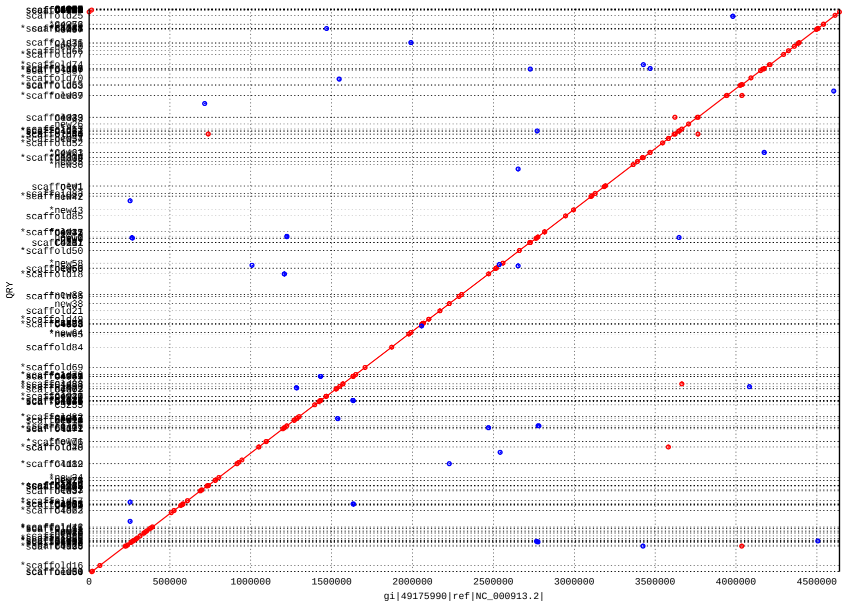

Figure 61: SOAPdenovo dotplot.

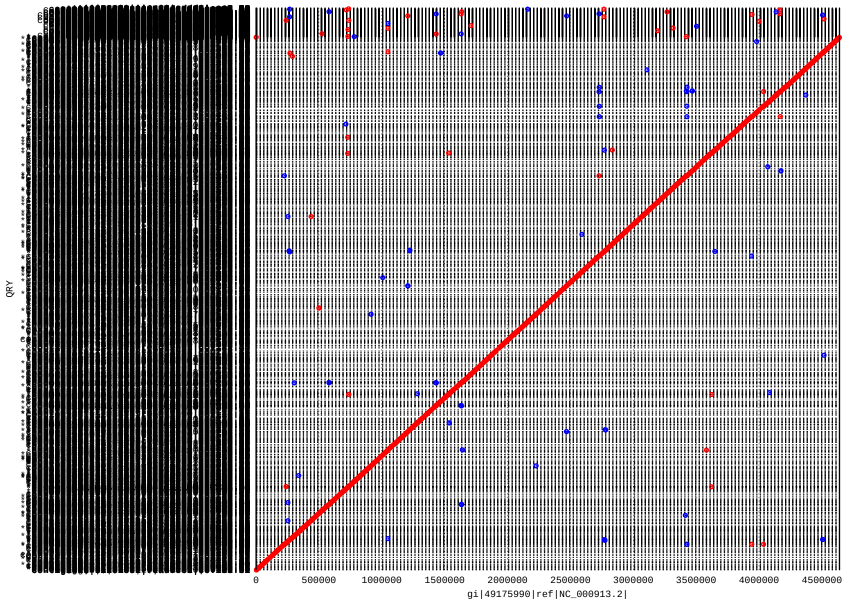

Figure 62: SSAKE dotplot.

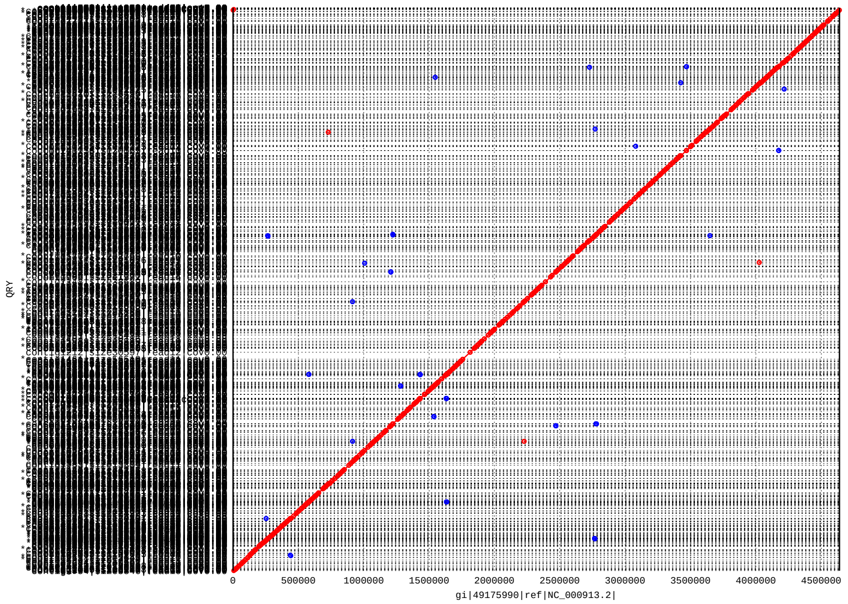

Figure 63: Taipan dotplot.

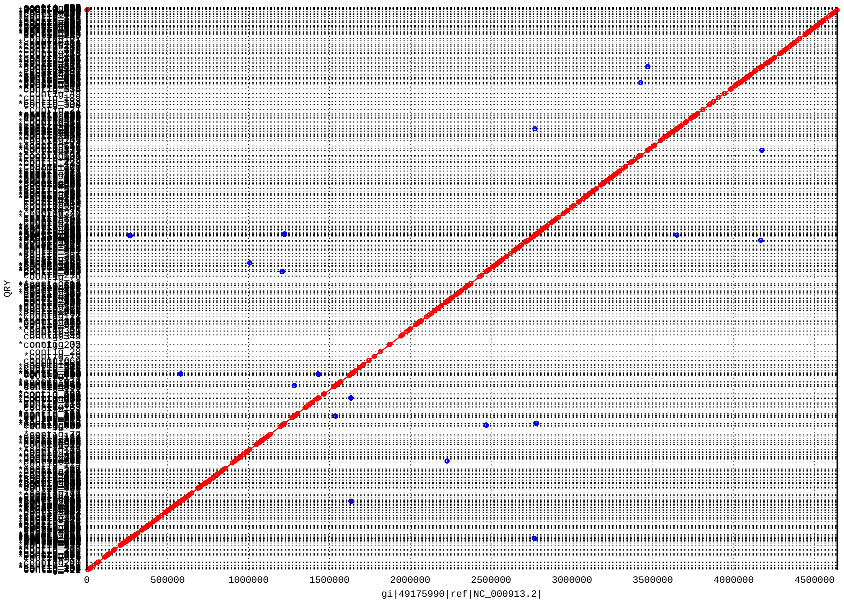

Figure 64: Edena dotplot.
