# Supplementary material for: Comparing De Novo Genome Assembly: The Long and Short of It
Source: PLoS One. 2011 Apr 29;6(4):e19175. doi: 10.1371/journal.pone.0019175 (PMC3084767; doi:10.1371/journal.pone.0019175)
Supplement: Table S1 — Parameter setting used for each assembler. (PDF) [file pone.0019175.s002.pdf]

Table S2: Short Read Assemblers parameter setting.

Assembler	*S. aureus*	*H. acininychis*	*E. coli*
ABySS	k=23	k=27	k=31 n=5
Edena	m=21	m=27	m=30
EULER-SR	k=21	k=27	k=28 CloneLength=215 CloneVar=40
SOAPdenovo	k =21	k=27	k=25 -R
SSAKE	default	default	m=17 o=4 r=0.7 t=1
SUTTA	k=21 W_{mp}=150 W_{de}=10 W_{bb}=140	k=27 W_{mp}=150 W_{de}=30 W_{bb}=140	k=29 W_{mp}=150 W_{de}=20 W_{bb}=140
Taipan	k=19 T=8	k=27 T=18	k=29 T=10
Velvet	k =21 cov_cutoff=7	k=27 cov_cutoff=8	k=29 ins_length=215 cov_cutoff=12 -exp_cov=24

NOTE: Long-read Assemblers have been ran with their default parameters.
